# New perspectives on Solid Earth Geology from Seismic Texture to Cooperative Inversion

**DOI:** 10.1038/s41598-019-50109-z

**Published:** 2019-10-14

**Authors:** Cuong Van Anh Le, Brett D. Harris, Andrew M. Pethick

**Affiliations:** 1 Curtin University, Western Australian School of Mines: Minerals, Energy and Chemical Engineering, Perth, Australia; 2 MinEx Cooperative Research Centre (MinEx CRC), Perth, Australia; 3 University of Science, Vietnam National University Ho Chi Minh City, Ho Chi Minh City, Vietnam

**Keywords:** Solid Earth sciences, Economic geology, Geophysics

## Abstract

Seismic and electromagnetic methods are fundamental to Solid Earth research and subsurface exploration. Acquisition cost reduction is making dense 3D application of these methods accessible to a broad range of geo-scientists. However, the challenge of extracting geological meaning remains. We develop the concept of “textural domaining” for 3D seismic reflectivity data. Dip-steered seismic texture attributes are combined with unsupervised learning to generate sets of volume rendered images accompanied by a seismic texture reference diagram. These methods have the potential to reveal geological and geotechnical properties that would otherwise remain hidden. Analysis of seismic texture presents particular value in hard-rock settings where changes in velocity may be negligible across rock volumes exhibiting significant changes in rock mass texture. We demonstrate application and value of textural domaining with three industry-scale field examples. The first example links seismic texture to rock type along a 400 km long transect through central Australia. The second and third examples partition dense 3D seismic data based on texture for complex hard rock terrains in Nevada, USA and Kevitsa, Finland. Finally, we demonstrate application of domaining within texture guided cooperative inversion of 3D seismic reflectivity and magnetotelluric data to provide new perspectives on Solid Earth geology.

## Introduction

Seismic reflection methods aim to map the distribution of true relative reflectivity, which can then be interpreted to recover the distribution of geological boundaries and structures^1^. Structures, ranging from salt bodies^2,3^ to conventional hydrocarbon seal and reservoir systems are routinely imaged in fantastic detail.

Advances in seismic technologies have been driven by both pure research and the economic imperative of finding energy sources, minerals, groundwater and geothermal resources. Within the hydrocarbon industry, seismic methods have evolved over the last century to a point where hydrocarbon bearing sedimentary horizons, just meters thick, are intersected thousands of meters below the surface^4–8^. Thereafter modern geo-steering while drilling based on real time sensing (e.g., electromagnetic sensing) can ensure precision drilling along the targeted reservoir. The tremendous success of seismic methods for hydrocarbon exploration and production is often due to the large acoustic impedance (i.e., the product velocity and density) contrast between shale seal rock and hydrocarbon bearing sandstones for conventional oil or gas plays. However, not all geological environments are as well suited to the traditional seismic workflows. For example, hard rock settings are often characterized by the absence of consistent coherent or continuous seismic reflectors.

Seismic reflection methods have recently been applied in the hard-rock environment, where they are starting to play a crucial role in delineating a wide range of mineral ore deposits^9,10^. Hard-rock environments may be inhabited by a diverse collection of cross-cutting irregular-shaped geological bodies like, granitic plutons, mineralized porphyries^11^, breccia’s, skarns, dykes, sills and alteration zones. Lithological, petrophysical, geochemical and geo-technical relationships are generally complex. We will explore the idea that these types of geological “objects” have potential to be revealed within seismic reflectivity imaging with unsupervised learning based on seismic textures distribution. Once identified, these objects can then feed into strategies for texture guided cooperative inversion of seismic and magnetotelluric (MT) data.

## Seismic Texture and Geology

Texture is a very ‘human property’. It may relate to any combination of shape, size, roughness, smoothness, orderliness, randomness, contrast, and homogeneity of an object or elements of an object. Rock texture is often associated with the specific nature, composition, or arrangement of mineral crystals. However, at a larger scale, rock mass may also be linked to a multitude of macroscopic textures associated with jointing, faulting, metamorphic processes, metasomatic processes or depositional/post-depositional processes in sediments^10,12–14^. Before considering 3D seismic textures in hard rock settings we’ll consider distribution of texture in a regional 2D seismic transect. Here we will demonstrate the connection between seismic texture and rock type below the vast interior of Australia.

**Figure 1:**
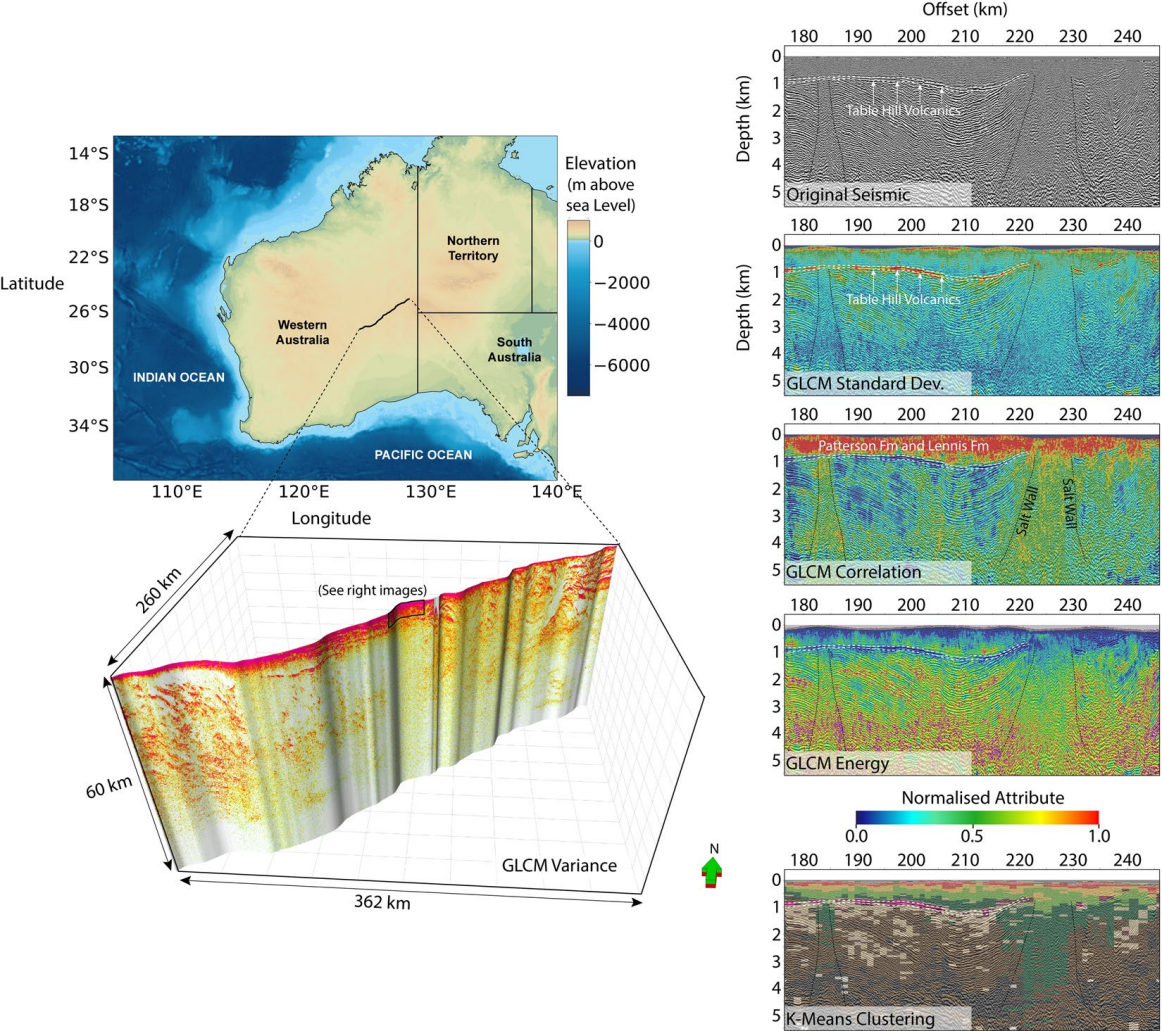
Representations of GLCM seismic textural attributes computed for the Yilgarn Block, Officer Basin, Musgrave Block (YOM) regional 2D seismic data across central Western Australia^15^. This shows extremes in seismic texture linked to (i) weakly consolidated sediments (ii) flood basalts and (iii) thick salt walls. Textural differences are effective in summarizing the large scale distribution of these major rock types. Significant differences in how well the GLCM seismic attributes are able to represent these different rock units are observed and so suspect that combination of attributes may be best for isolating volumes with common rock mass characteristics. It is these extremes in seismic texture that inspires us to consider methods for geo-statistical domaining based on seismic texture for high resolution 3D seismic data collected in hard rock settings. The location map displays elevation data sourced from the NOAA ETOPO1 global relief model^72^, generated using Python 2.7^73^ and assembled with Microsoft Visio 2016^74^.

The Yilgarn Craton - Officer Basin - Musgrave Province (YOM) regional 2D seismic transect was acquired along more than 400 km of the Great Central road, which passes through Central Australia^15^. Its location is provided at the top left of Fig. 1. A Gray-Level Co-occurrence Matrix (GLCM) seismic texture attribute image (i.e. variance) for the full transect is located the bottom left of Fig. 1. The YOM seismic data is publicly available^15^ and a general description of GLCM seismic texture attributes^16–19^ is provided in our methods section.

To the right of Fig. 1 the GLCM seismic texture attribute images for standard deviation, correlation and energy can be compared with the seismic reflectivity imaging within a 70 km long, 5 km deep window. While no new information can be created by a seismic attribute, “hidden” objects can be revealed. The range of values for the GLCM texture attributes are normalized between zero to one^16–19^. A cosine of phase attribute^20^ is co-rendered as an intensity overlay on the attribute images to the right of Fig. 1.

Inspection of the GLCM seismic texture attribute images in Fig. 1 suggest links to regional scale geological objects which span hundreds of kilometers. The most prominent are marked on Fig. 1 and include; (i) shallow clastic sediments of the Patterson and Lennis Fm, (ii) the Table Hill Volcanics and (iii) sub vertical salt walls. We also observe that these large objects are not equally well characterized by the different textural attributes. For example, in the right hand panels in Fig. 1 we see that:

(i) The expansive shallower more weakly consolidated clastic sediments that make up the Patterson Fm and Lennis Fm are strongly represented by a relatively high GLCM correlation texture attribute,
(ii) The expansive flood basalts that are the Table Hill Volcanics appear as a relative high in the GLCM standard deviation and a relative low in the GLCM energy attribute image,
(iii) The thick salt walls that have pushed through the high reflectivity layering of the Neoproterozoic strata below the Table Hill Volcanics, have relatively high values for the GLCM correlation seismic attribute.

While there appears to be a link between seismic textures and regional geology, no single seismic texture attribute offers sufficient clarity to differentiate these large scale geological objects. Unsupervised learning based on combinations of seismic texture attributes could generate improved, perhaps automatic, delineation of these large rock packages within the YOM seismic image.

**Figure 2:**
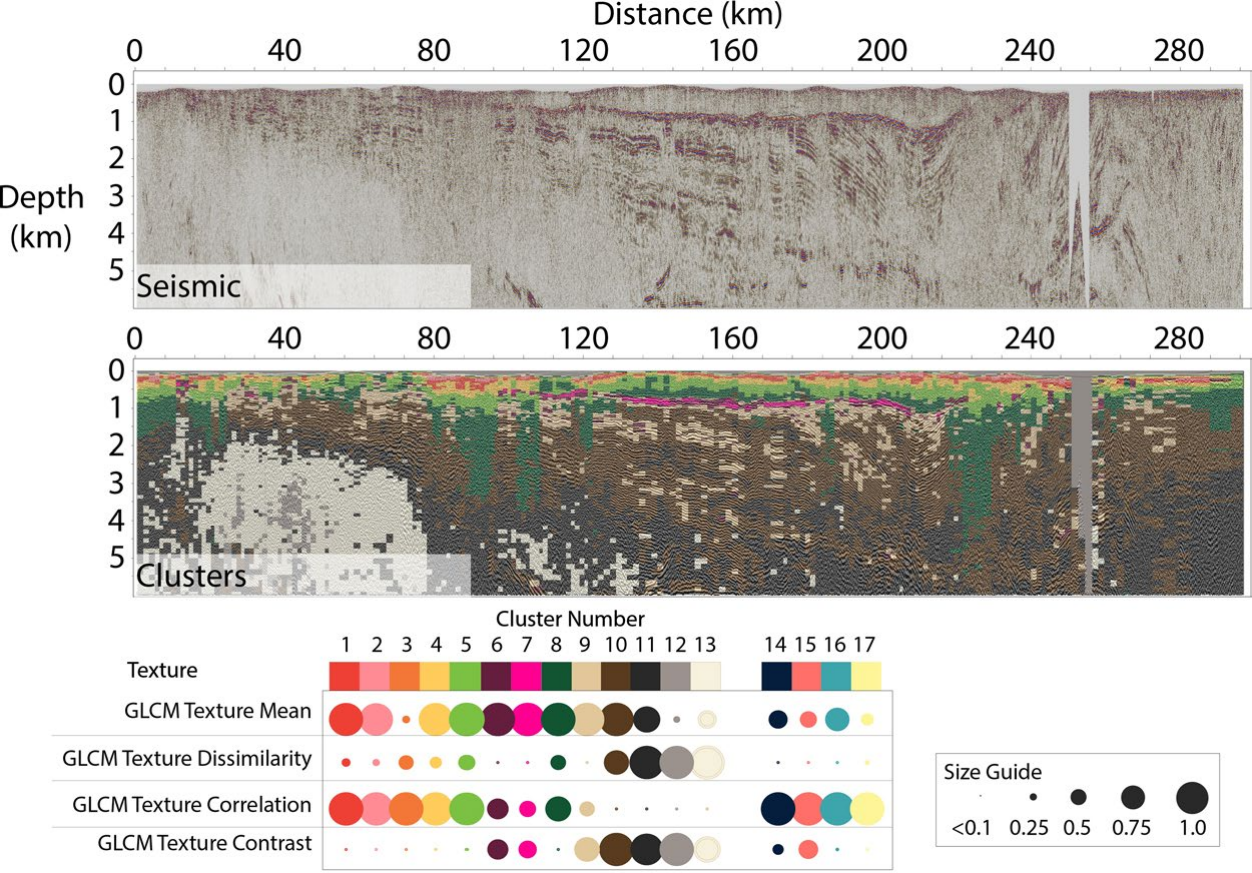
A 320 km subsection of (top) 2D reflection seismic and (bottom) K-means textural clustering of four GLCM seismic texture attributes. Clustering was performed using GLCM textural attributes, mean, dissimilarity, correlation and contrast using 17 different clusters. Clusters 14 to 17 are outliers, representing combinations of attributes that do not represent any meaningful geological features. The relative contribution of each normalized GLCM attributes to each cluster is represented by a dot matrix diagram and associated size guide. While not perfect, the major geological divisions are reasonably well represented. The Table Hill Volcanics can be linked to clusters 6 and 7 and Salt Walls to cluster 8. The clustering provides a valuable aid to interpretation.

We deploy K-means clustering^21–27^ (described in the methods section) with the GLCM seismic texture attributes dissimilarity, mean, contrast and correlation as input. Figure 2 provides the original seismic image and the resulting cluster distribution. The cluster distribution has clear associations with; (i) the Lennis Fm and the Patterson Fm (i.e. dominated by clusters 1 to 5), (ii) the salt walls (vertical structures associated with cluster 8), (iii) the Table Hill Volcanics (i.e. dominated by clusters 6 and 7), (iv) Neo-Proterozoic sediments below the Table Hill volcanics (i.e. dominated by clusters 9 and 10) and (v) rocks below the Neo-Proterozoic sediments (i.e. dominated by clusters 11 and 12).

Other clusters (14 to 17) contained textural outliers with an insignificant number of cells. Cluster 12 identifies null traces where seismic coverage was insufficient for imaging. While not perfect, the K-means clustering with GLCM texture attributes as input (i.e. textural domaining) generates clear links between clusters and large-scale geology. This clustering strategy provides assistance to geological interpretation from seismic imaging that spans more than 300 km. From the relatively large number of clusters we can readily connect clusters to geology.

The example in Fig. 2 presents the outcome from one seismic texture domaining workflow. That outcome depends on selection of input seismic attributes and clustering parameters such as cluster number. Selection of input seismic attributes and clustering parameters should be based on the nature of the reflectivity data and specific objectives assigned to the textural domaining.

While our 2D example for the YOM transect of central Australia serves as motivation to proceed, we do recognized that 2D seismic imaging presents limitations such as out-of-plane seismic reflectors. These are particularly problematic in hard rock settings where sub-vertical geology is common.

We will now focus on “3D seismic texture domaining” for hard rock settings. These settings are often characterized by high velocities and a lack of consistent or coherent reflectivity. For hard rock settings, distribution of seismic texture could be a pathway to revealing valuable relationships between 3D seismic reflectivity data and geology. We’ll use modern high resolution 3D seismic reflectivity datasets from Nevada (USA) and Kevitsa (Finland) as examples.

## 3D “Seismic Textural Domaining” in Hard Rock Settings

In hard rock settings it is possible or even common adjacent rock volumes to have similar average acoustic velocity^28,29^ but dramatically different seismic and rock texture. Discriminating between such volumes can be critical, especially when exploring or mining economic ores. We show how “seismic texture domaining” can be achieved with a combination of dip-steered seismic attributes and unsupervised learning.

Seismic attributes are extensively used in the hydrocarbon industry. For example they routinely provide indications of 4D reservoir fluid movements in time and space^30,31^. A comprehensive table of applications for seismic attributes is provided by Roden, R., Smith, T. and Sacrey, D^32^.

The techniques we develop can classify domains with common seismic texture based on dip-steered textural attributes and statistical clustering. For our 3D examples, a K-means clustering result is generated from the four GLCM textural attributes, energy, entropy, homogeneity, and contrast^16–19,33,34^. The outcome is rendered with volumetric ray casting, revealing regions of common seismic texture. Computational details for our seismic texture domaining are included in the Methods section.

Three examples are provided to show the application and value of “textural domaining”. These focus on (i) characterization of deep cover and basement at a site in Nevada USA, (ii) rock mass characterization at the Kevitsa mine in Finland, and (iii) textural domains as input to seismic texture guided 3D cooperative inversion of seismic and magnetotelluric data. Background on each site is provided as supplementary information (see Supplementary Information).

**Figure 3:**
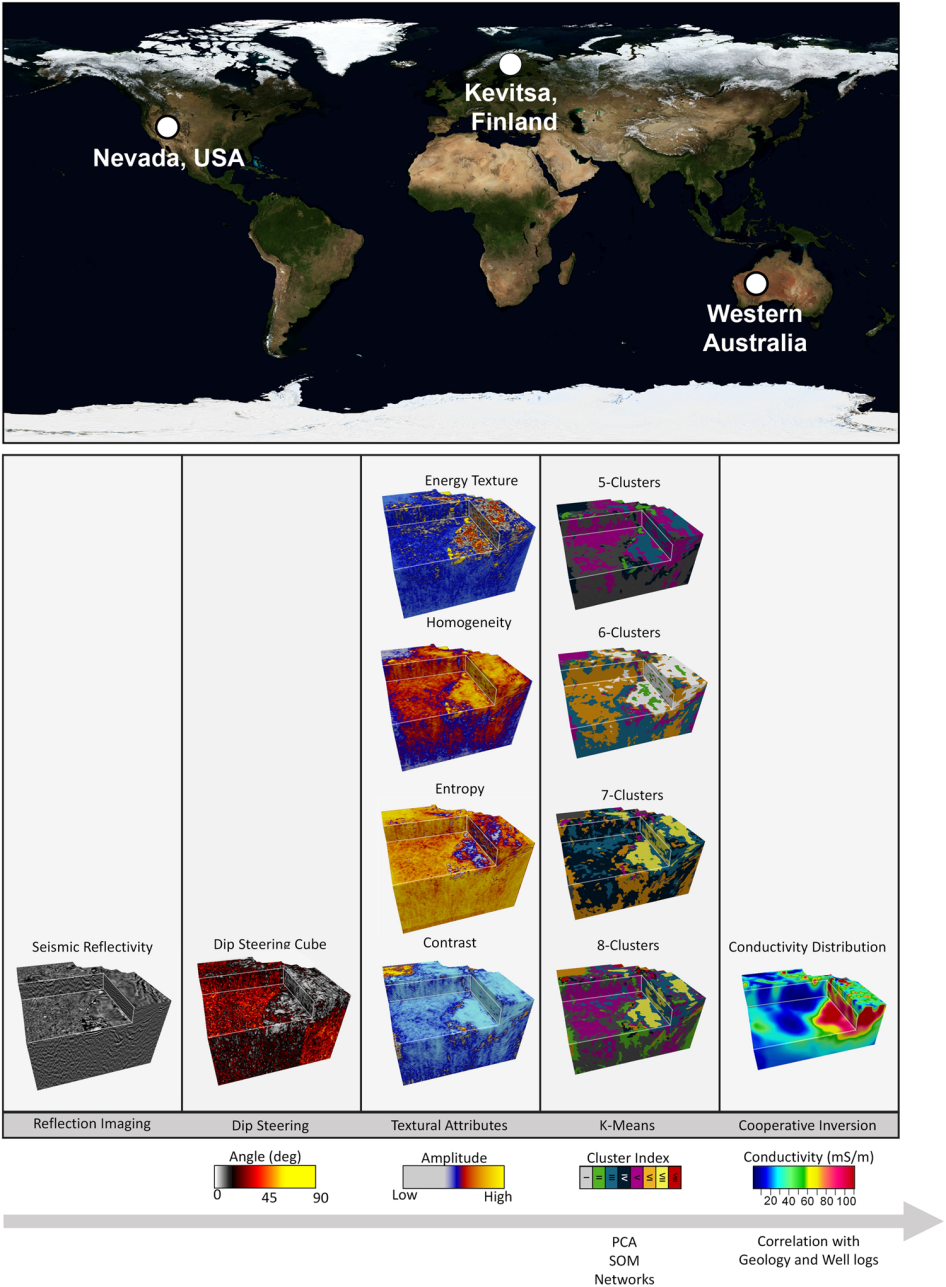
World map (modified from Blue Marble W/Topograpy^75^) showing the location of the 3D seismic surveys from the Carlin gold district in Nevada, USA, the polymetallic Kevitsa mine site in Finland and the long 2D seismic YOM transect^15^, Western Australian (upper image) along with a graphic overview of major steps, inputs and outcomes for seismic texture domaining (lower image). For the overview each 3D image is an approximately 5 km by 5 km by 2 km cube. The reflectivity image is post-stack seismic data. The schematic provides the steps required to take the Nevada seismic reflectivity volume, from dip-steered textural attributes, to the outcome of K-means analysis for 5, 6, 7 and 8 clusters, and finally to an electrical conductivity distribution derived from cooperative inversion. While we represent multiple outcomes from K-means analysis, methods like principle component analysis (PCA), self-organising maps (SOM) and deep learning may all have value in revealing geological objects hidden within 3D seismic reflectivity imaging.

Figure 3 shows the test site locations and a diagram that graphically illustrates the inputs and outputs needed for our volumetric domaining based on seismic texture. The volume rendered images in Fig. 3 are for a 5 km by 5 km by 2 km cube at the Nevada site. This diagram provides a high-level view of our conversion of 3D seismic reflectivity data to domains with common seismic texture. It also represents an outcome from the rapidly evolving discipline of cooperative inversion^35,36^ (also see Supplementary Information: S2. MT Inversion and Cooperative Inversion). The ModEM3D^37,38^ unconstrained electrical conductivity result is also shown.

As indicated in Fig. 3, there are alternative numerical pathways for quantitative extraction of domains based on seismic attributes derived from a reflectivity volume. Reasonable candidates might include; principle component analysis (PCA^32,39^), self-organizing maps (SOM^32,40^), fuzzy clustering analysis^41,42^ or modern machine learning combined with neural networks, also referred to as deep learning^43^. Depending on circumstances and objectives, each may present value could conceivably be worked into a textural domaining workflow. We have chosen to integrate K-means clustering to illustrate seismic textural domaining methods. K-means algorithms are well tested and understood within geophysics^21–27^ and geology^44,45^. K-means clustering fits in the broader category of Partitioning Relocation methods. Berkhin^46^ assesses an extensive range of clustering methods which he describes as essentially “*unsupervised learning of a hidden data object*”.

The impact of statistical outliers is identified as a potential problem for K-means methods^42^. However, if high resolution 3D seismic data is correctly processed then such outlines should be rare. That is, processing collects seismic traces from a large number of geophone-source combinations (e.g., offsets and azimuths) to yield the final reflectivity image (i.e., often stacking then migration). Another advantage of K-means cluster analysis is that it generates a set of centroids or means that help summarize the nature of each cluster with reference to the inputs. These give statistical and geometric meaning to each cluster. In summary K-means clustering tends to be a reasonable base line method that should be able to reveal 3D geological objects hidden deep within modern 3D seismic reflectivity data sets, which can be composed of billions of data points.

K-means analysis requires selection of a number of clusters. Cluster validation measures^47^ provide some sense of the number of clusters that might be statistically and hopefully physically meaningful and we will discuss these in the examples below. Figure 3 points towards an early and relatively simple conclusion. That is, modern parallel computing permits us to generate and compare outcomes for different combination of attribute or cluster numbers with little additional effort. For example in Fig. 3 we represent textural domaining outcomes for the Nevada reflectivity data for 5, 6, 7, and 8 clusters. If the purpose of the textural domaining is to provide the simplest possible division between cover and basement as input to potential field or EM inversion then 5 clusters may be sufficient. If extremes in texture are of interest then a higher number of clusters would need to be selected to identify subtly perhaps hidden in the imaging. At a base level, textural domaining acts as an aid to interpretation of 3D seismic data. At a higher level, textural domaining may be used to identify a specific target hidden within a mass of 3D reflectivity data.

There are many examples of the effective application of k-means clustering in geology and geophysics^21–27^. Di, *et al*.^48^ suggest an interpreter-guided initialization of k-means clustering to preferentially define salt-dome targets. We have not biased our application of k-means clustering, although we appreciate that there may be alternative methods for unsupervised learning where a specific target is pre-defined. If sufficient reliable prior information exists, then it is conceivable that Deep Neural Networks (DNN) can be combined with unsupervised learning as described in Yang, *et al*.^49^.

In the below examples we will illustrate the potential of textural domaining. One example is from the Calin Gold district in Nevada USA and the other is from the polymetallic Kevitsa mine in Finland.

All computational details on how seismic texture domaining is achieved are provide in the Methods sections (Methods).

### Example 1: Seismic texture domains in cover and basement rock, Nevada USA

In our first example we work through the main steps and outcomes for textural domaining of a hard rock 3D seismic data set from the Carlin gold district of Nevada USA. The site passes across a major fault with significant displacement of cover sequences that are between 100 and 500 m thick. Details of the the Nevada site and datasets are provided in the supplementary information.

Partitioning of the seismic reflectivity volume into a finite number of domains (or clusters) with common seismic texture is achieved with K-means analysis (see Methods). For K-means analysis it is possible to generate outcomes with any number of clusters; however, elements of the outcome may fail to have value if the number of clusters is too high or low.

Internal cluster validation measures^47^ provide a possible rational for determining the optimal number of clusters. They are statistical guides based on coherences within clusters and distances between cluster sub volumes. Specifically, we have employed the Davies-Bouldin and Calinski-Harabsz validation measures^47^ to help choose the number of clusters as described in the section, Methods. These suggest that between 3 and 10 clusters would be reasonable for the Nevada data set (also see Fig. 3 for outcomes with 5, 6, 7, and 8 clusters). We have elected to explore a seven-cluster image and develop an accompanying seven cluster seismic texture reference diagram. In selecting seven clusters we are partly guided by the validation measures and partly by the recognition of at least three significant seismic textural domains within basement and three within cover rock for the Nevada reflectivity volume.

Once the number of clusters is selected, the mean values of the centroids from the clustering, based on the four input seismic texture attributes can be extracted and plotted. This is graphically expressed as a “seven-cluster seismic texture reference diagram” which provides the seismic texture combinations that characterize each cluster. Some clusters will map out a distinct rock mass while other sets of clusters can be more gradational in nature.

**Figure 4:**
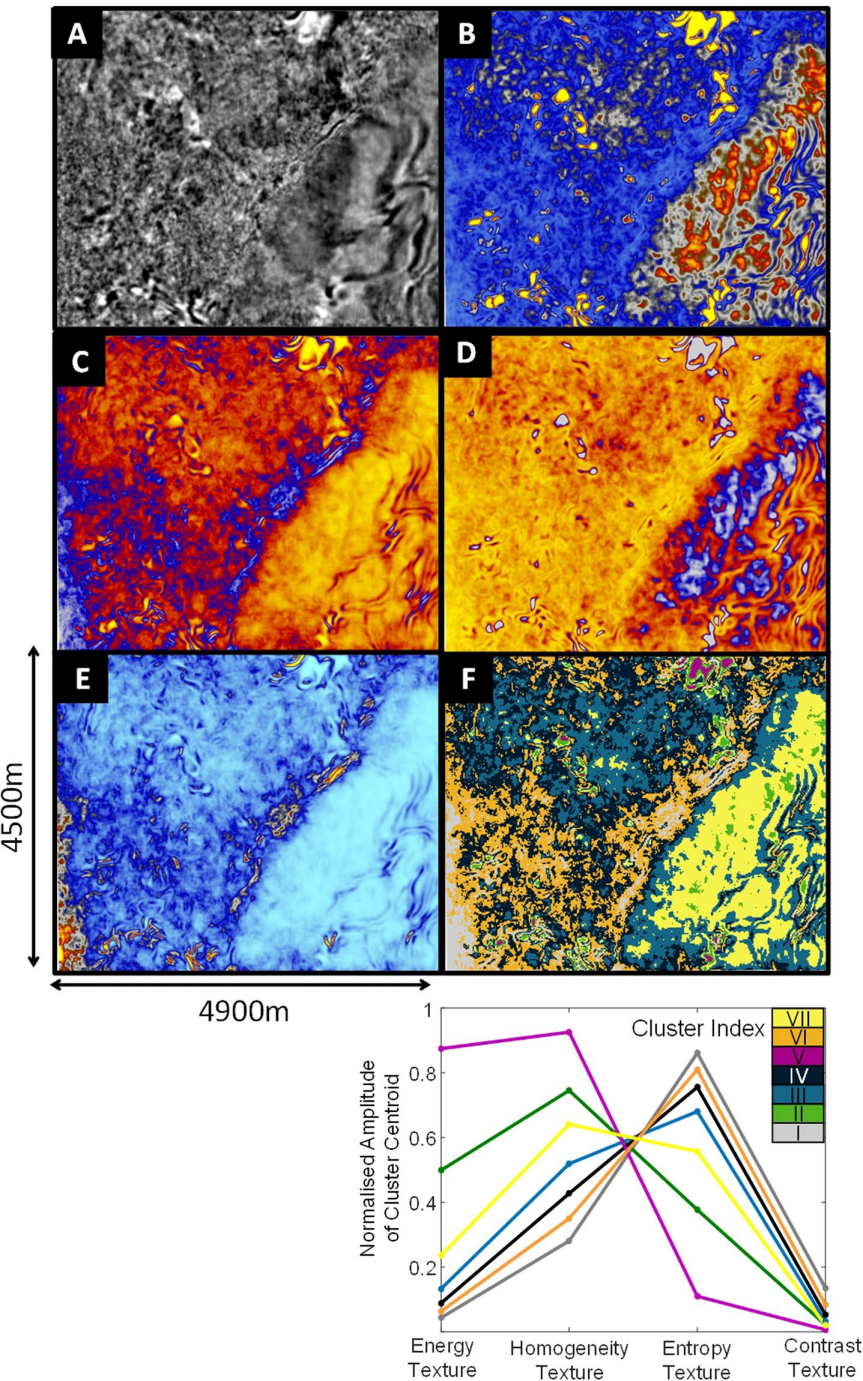
Plan view slices extracted from the Nevada seismic volumes at 400 m below ground level for (**A**) Seismic reflectivity, (**B**) energy texture, (**C**) homogeneity texture, (**D**) entropy texture, (**E**) contrast texture and (**F**) seven cluster seismic texture image. Each cluster is characterized by distribution centroids across the input textures. We call the diagram at the base of the images a “seven cluster seismic texture reference diagram”. This diagram provides a way to describe the each cluster and shows relationship between clusters (e.g., are clusters distinct or gradational).

Figure 4 provides depth slice images at 400 m below ground level for the seismic reflectivity image (Fig. 4A), energy texture (Fig. 4B), homogeneity texture (Fig. 4C), entropy texture (Fig. 4D), contrast texture (Fig. 4E), seven cluster seismic texture cluster image (Fig. 4F), and the seven-cluster seismic texture reference diagram (Fig. 4). The clusters shown in the reference diagram (Fig. 4), and seismic texture domain image (Fig. 4F) are color-matched. Color matching allows the combinations of seismic textures that dominantly contribute to each cluster to be identified.

**Figure 5:**
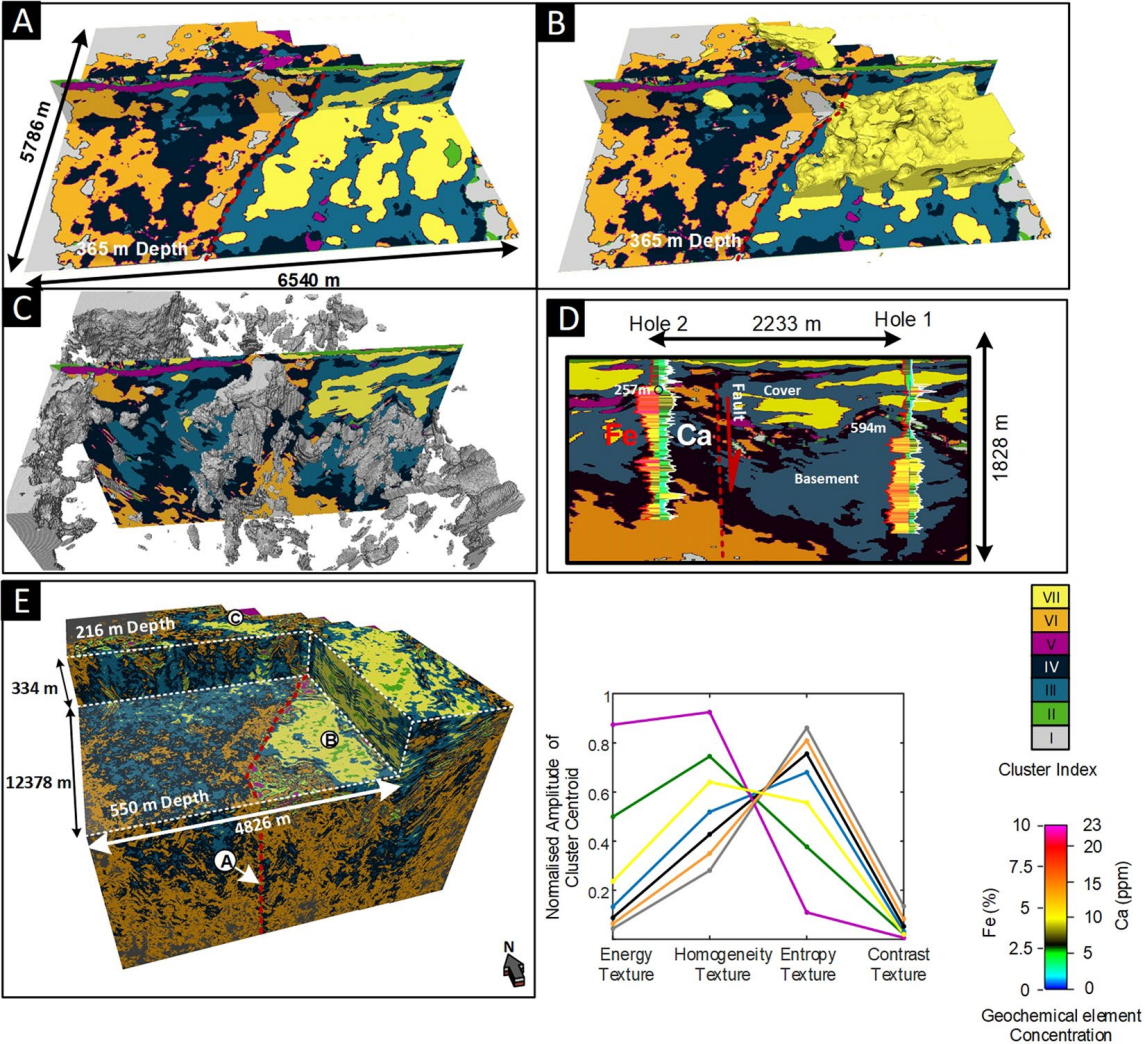
Volume rendered^57^ 3D images from seismic textural domaining of the Nevada USA reflectivity data. (**A**) A crossline and plan-view section extracted from the filtered 3D cluster volume at 365 m depth. (**B**) A 3D rendering of the Cluster VII (yellow), which relates to a distinct group of sediments in cover. (**C**) A 3D rendering of Cluster I (gray) that maps out narrow sub-volumes nested along the major fault. (**D**) A cross section view through drill holes 1 and 2 showing distribution of Fe and Ca relative to the seismic textural clusters. (**E**) Volume of the seven-cluster seismic texture domaining image with cut-out. The relationship between seismic texture of each of the seven clusters is shown as the seven-cluster seismic texture reference diagram. The major fault is down-thrown to the right of the images and Clusters III, IV and VI dominate the basement rock. The approximate trace of the fault plane is marked as the red dashed line in (**A, B, E**). Depth to basement is 257 m for Hole 1 and 594 m for Hole 2.

The depth slice in Fig. 4F immediately reveals the transition from Paleozoic basement rock to younger cover rock, which is dominated by cluster VII (yellow). However, the value of textural domaining lies with volumetric analysis. Figure 5 provides several representations of the 3D distribution of textual domains along with a comparison with geochemical analysis (calcium (Ca) and iron (Fe)) obtained from cores in two drillholes.

For Fig. 5A–D we have completed an additional processing step. A volume statistics filter that replaces the center value of a sliding 3D calculation window with the most frequent occurrence value is applied^50^. We use a calculation window of five inline and five crossline traces, with a depth gate of 15 m.

Figure 5D provides a vertical section showing seismic texture domains. Overlain on the section are two drillholes showing vertical distribution of Fe and Ca concentration. Large changes in Fe and Ca concentration can be mapped to major and characteristic changes in textural domains hinting at a connection between cluster distribution, geological environments and geological processes. For example, a sharp increase in both Fe at the transition from cover into basement rock at approximately 257 m in Hole 2 and at 594 m in Hole 1. Both geochemistry and distribution textural domains can assist interpretation of basement geometry beyond a more complex, transitional zone.

In Fig. 5E, point B identifies the main thick package of cover sequences on the down thrown side of the major fault. The point marked C in Fig. 5E locates a channel like structure on the upper fault block. The channel or basement depression runs towards the edge of the major fault. We will see later that mapping the volumetric distribution of cover rock on each side of the major fault is a significant input necessary for successful cooperative inversion of seismic and magnetotelluric data (see Example 3).

In Fig. 5, Cluster VII (yellow) is distinct and simultaneously exhibits medium homogeneity and entropy texture with low energy and contrast GLCM seismic textures. Cluster VII is an expansive seismic texture domain that has a strong link to the shallow dipping electrically conductive packages of rock within the cover. Cluster VII is almost exclusively found within the cover sequences.

In contrast to Cluster VII (yellow), Cluster I (gray) characterizes volumes with the highest entropy and contrast texture with lowest homogeneity and energy texture centroids. Cluster I (gray) could be considered as the extreme of the volumetrically more expansive Cluster VI (orange). However we observe that cluster 1 does have a focused spatial association with transitions across the major fault (i.e. see the red dashed line of Fig. 5A,B) and from cover to basement rock (i.e. see the grey dashed line in Fig. 5D). These patches and zones of combined high seismic entropy and contrast identified along specific corridors may be of interest to mineral explorers. This example has identified spatial groupings of texturally similar objects hidden within a mass of seismic reflectivity data.

Cluster V (magenta), maps out a textural domain characterized by high energy and homogeneity texture combined with low entropy and contrast texture centroids. Cluster I (gray) and Cluster V (magenta) are distinct textural extremes that are almost never spatially associated.

Seismic texture domaining can assist in interpretation of subtle textural changes across and along faults. There are several examples in the Nevada images. For example point A in Fig. 5E identifies vertical feature likely to be associated subtle changes in texture across a major fault plane within the basement rock (i.e. see red dashed line in Fig. 5E). We see again that unsupervised learning provides support to interpretation of subtle changes in textures within hard rock reflectivity data.

Some seismic texture domains are almost exclusive to cover or basement for the Nevada example (e.g., Cluster VII); however, others (see Clusters III or IV in Fig. 4) are represented in both cover and basement. This is not unreasonable as expansive packages of volcanic rock are indeed found in both the cover and the Paleozoic basement rock. More generally it’s incorrect to impose an expectation that textural domaining should provide a direct map of solid geology. What it does offer is a tool to significantly improve volumetric geological interpretation of 3D seismic imaging. It has the capacity to map out volumes with seismic texture combinations potentially related to types of rock mass that may otherwise remain hidden.

### Example 2: Seismic texture and rock mass characterization; Kevitsa Finland

The polymetallic Kevitsa ore deposit contains copper (Cu), nickel (Ni), and platinum group elements (PGE)^10^. It is expected to contain approximately 240 million tons of nickel using a cut-off grade of approximately 0.1%^10^. The host intrusion varies from gabbro to dunite composition. It is characterized by distinct magmatic pulses likely to be responsible for these different phases. The Kevitsa 3D seismic survey was completed over what is now an active mine. The geology below the Kevitsa mine site is strongly 3D and in places the polymetallic ore zone is crosscut by the prevailing seismic dip^10,51^— this presents as a highly challenging data set. Details and references for the Kevitsa mine site geology and seismic survey (processing and acquisition) are provided as supplementary information in Supplementary Information (S1.2. Kevitsa Finland) and Le, *et al*.^52^.

The same steps taken to create seismic texture domains for the Nevada seismic reflectivity image have been followed for the Kevitsa dataset. K-means cluster analysis is tested for a range of total number of cluster centroids, spanning from 2 to 11. As with the Nevada data set, the Davies-Bouldin and Calinski-Harabasz cluster validation measures are computed. When viewed together they suggest anywhere between 5 and 10 clusters may be reasonable. For Kevitsa, we examine images with 10 clusters, which is three additional clusters compared to the number used for the Nevada example. We select a higher number of clusters to examine the potential influence of low signal-to-noise zones on the margins of the seismic data coverage. That is, we deliberately include very low-fold seismic data at the north of the seismic image and examine how potentially lower quality imaging (e.g., low or inconsistent seismic fold) may present in textural domaining.

**Figure 6:**
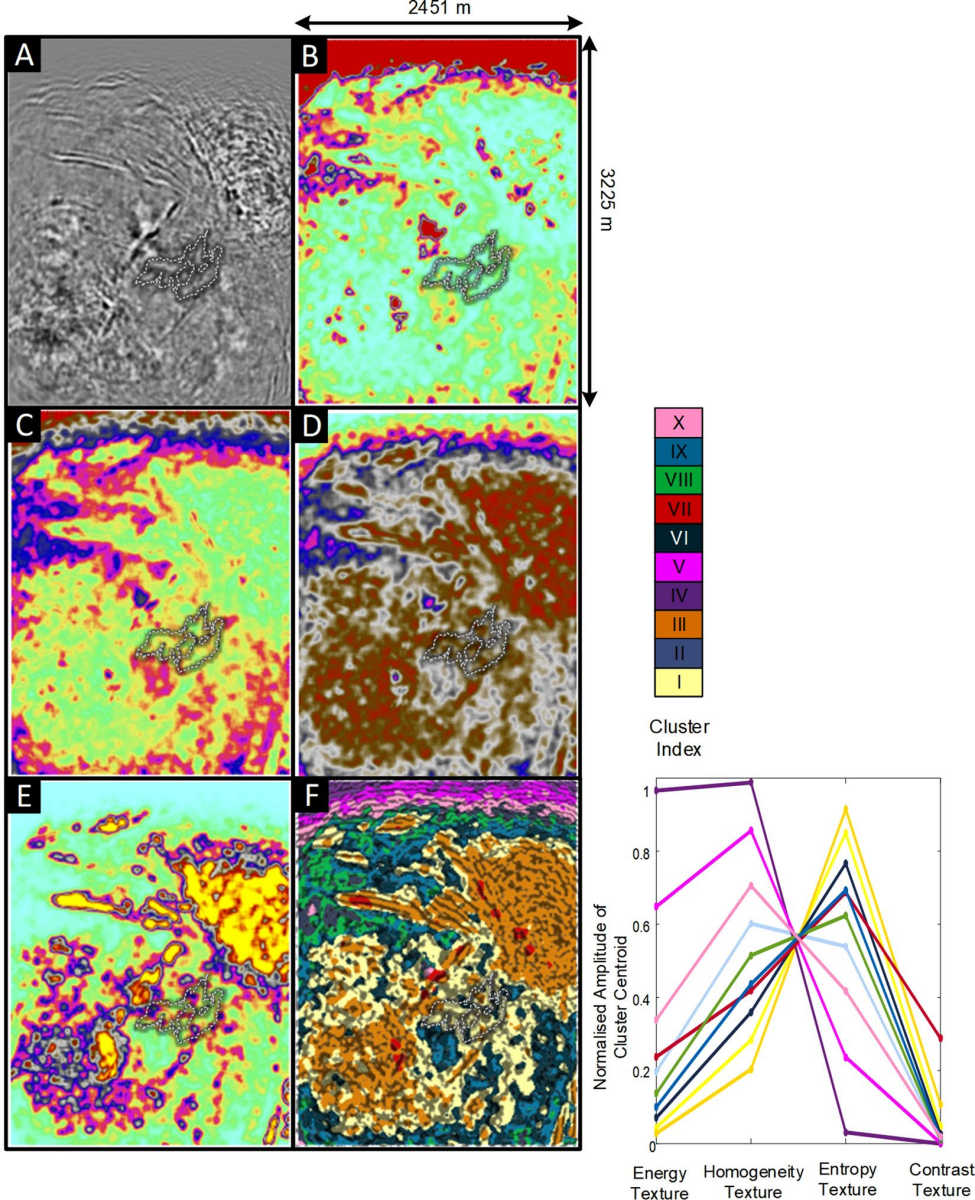
Comparison of the seismic reflectivity, GLCM texture seismic attributes and textural clustering at a depth slice 500 m below ground level for the Kevitsa seismic volume in Finland. The image includes: (**A**) Seismic reflectivity, (**B**) energy texture, (**C**) homogeneity texture, (**D**) entropy texture, (**E**) contrast texture, and cluster indexes. The dotted white line shows the contour of 0.15% Ni concentration. (**F**) The seismic textural domains overlaid by the seismic attribute cosine of phase. The relationship between seismic texture of each of the ten seismic textural clusters at the Kevitsa site is provided.

Figure 6 shows depth slice images at 500 m below ground level for the processed Kevitsa seismic reflectivity data^10^. It includes the four GLCM seismic texture attribute images (Fig. 6B–E), a horizontal slice through the seismic textural domains Fig. 6F) and the associated 10-cluster seismic texture reference diagram. Superimposed on each panel is a 0.15% Ni cut-off contour to broadly represent the shape of the Kevitsa Ni mineralization. The images of seismic textures are provided with an intensity overlay of the cosine of phase seismic attribute which gives a detailed impression of the seismic reflectivity distribution.

**Figure 7:**
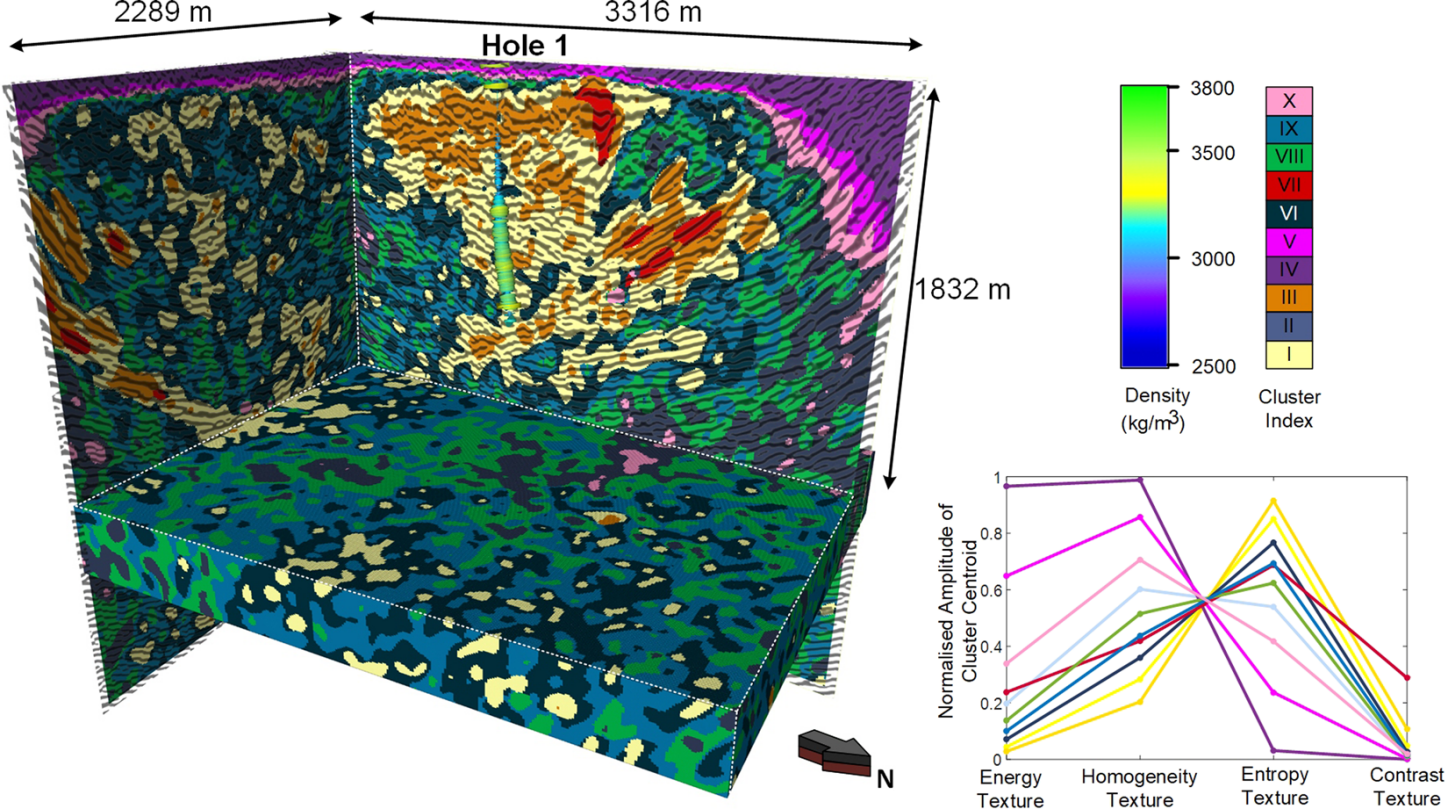
A cut-away 3D image of the Kevitsa seismic textural domains with an accompanying 10-cluster reference diagram. Overlying the data is a density well log from Hole 1. Several observations can be made in relation to the distribution of each cluster. Clusters IV, V and X represent anomalous textures at the fringes of quality imaging. Textural domaining provides volumetric information from which anomalous or zones of interest are quickly identified.

**Figure 8:**
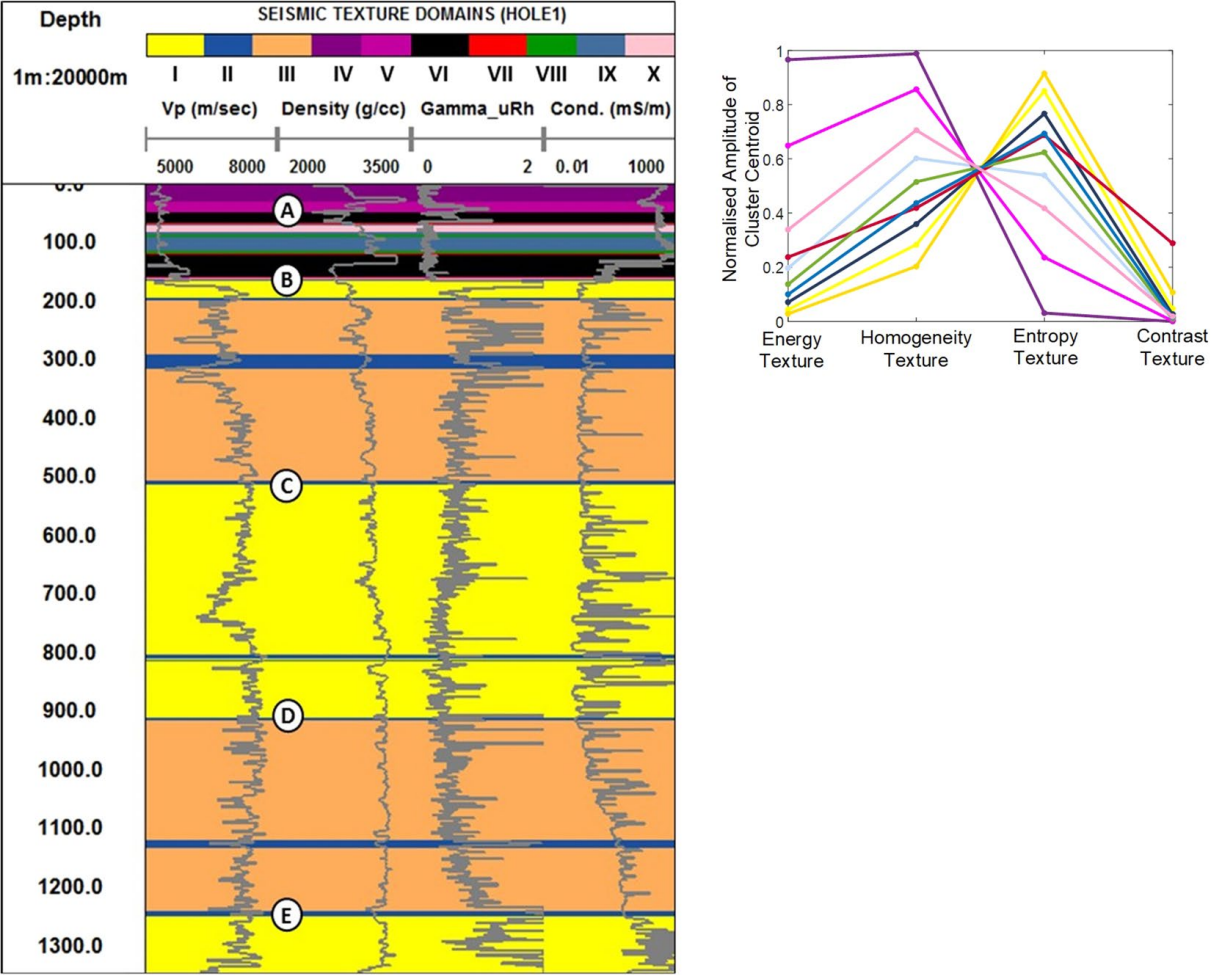
Stripe logs showing velocity, density, gamma and conductivity over textural cluster distribution extracted along the trace of Kevitsa drillhole 1. To the right of the stripe logs is the Kevitsa 10 cluster seismic texture reference diagram. Points A to E highlight key transitions in both textural clusters and the character of the petrophysical logs. Points A and B mark major changes in texture associated with changes in the character of gamma, density and conductivity logs. Points C, D, and E are associated with more subtle changes between closely related clusters 1 (yellow) and 3 (orange) and the overall character of several petrophysical logs between the boundaries. For example, a significant change in conductivity and gamma occurs at point E, although the character of velocity and density don’t appear to change significantly along the trace of drillhole. The gamma and conductivity may indicate the rock is different and the transition from cluster 3 to cluster 1 appears to independently support this. Note that there is no justification for asserting a causal relationship between the gamma, conductivity and the textural clusters.

**Figure 9:**
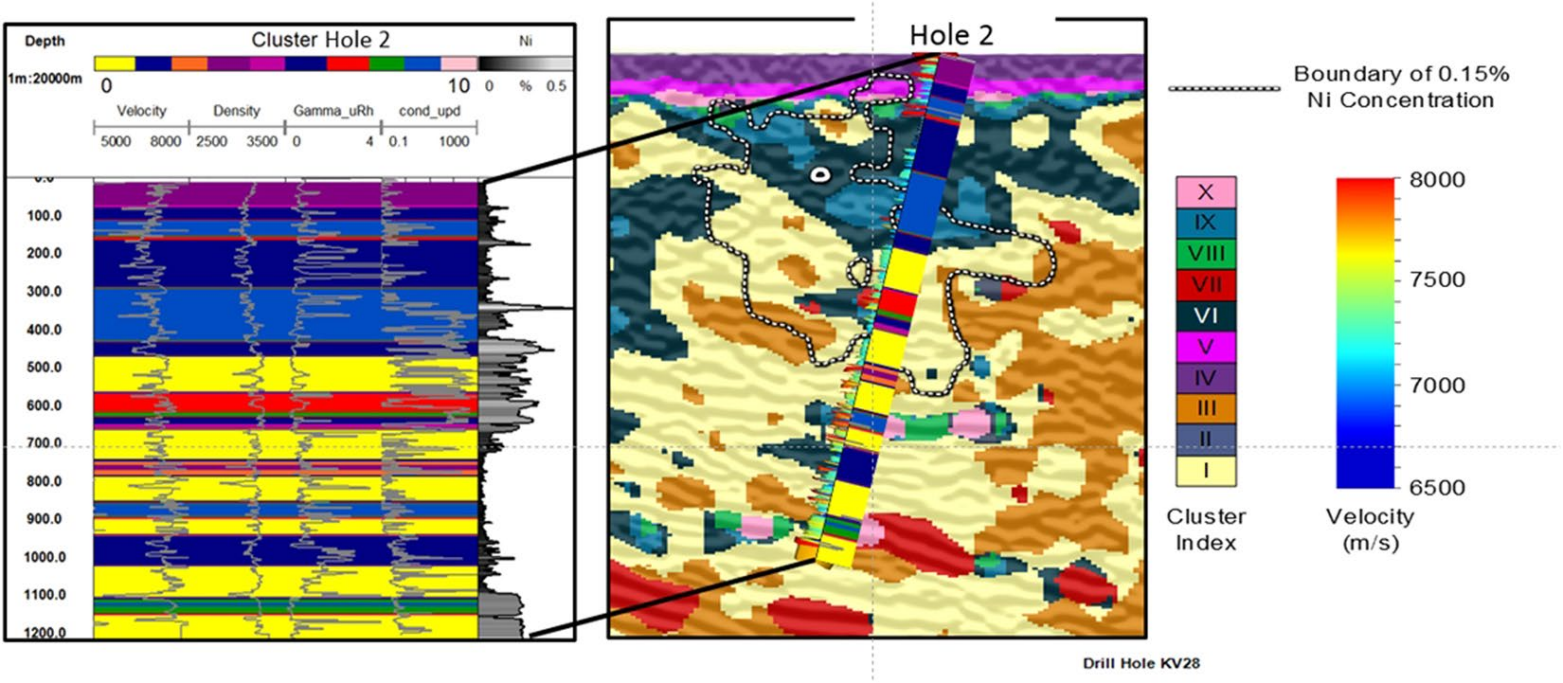
Images showing wireline logs plotted^76^ over color coded seismic textural cluster distribution along the trace of the Kevitsa drillhole 2. The image includes plots of velocity, density, gamma and conductivity along with Ni percent. Point A marks the change from cluster I which has the second highest textural entropy centroid. Point A also coincides with a change from lower to higher velocity and density. The petrophysics in Kevitsa drillhole 2 is more complex than that for Kevitsa drillhole 1 where there is negligible mineralization (barren rock). That is, high Ni and high conductivity are not bound within one particular textural cluster.

The northern limit of the images is characterized by gradation from Clusters IV (purple) to V (magenta) to X (rose). These clusters tend to map out the anomalous textural extremes associated with areas of progressively low seismic fold towards the northern extreme of the image (i.e., low or inconsistent coverage of seismic source and receiver combinations were included to the North only). Note that the textural combination represented by cluster X (rose) do occur within the high and uniform fold seismic coverage. Later we will directly compare the seismic texture clusters with petrophysical data collected along the trace of drillholes (e.g., see Figs 7, 8 and 9).

In Fig. 6, the interior of the 0.15% Ni shell is dominantly Cluster I (yellow). Little of Cluster II (gray-blue) or Cluster III (orange) is associated with the ore shell for the depth slice at 500 m below ground level. There are also near linear bounding zones for Clusters III (orange) and VII (red) to the north-west and south-east of 0.15% Ni shell. The ore shell also appears to be bounded by Clusters IX (Turquoise) to the south-west. This observation indicates that the cluster distribution at the 500 m depth slice provides some geometric definition for aspects of the 0.15% Ni shell. That is, Textural Domaining has found potentially hidden geometries within a complex 3D seismic volume that appear to have some connection to the distribution of Ni ore.

The relationships between Ore and seismic texture are complicated by the multiple phases of emplacement of Cu, Ni, and PGE which can crosscut major stratigraphic dips^10^. While no cluster uniquely matches the Ni shell, there are credible geometric associations between the distribution of clusters and the ore shell. A volume rendered image in the supplementary information (see Fig. S1) illustrates how Cluster III (orange) appears to wrap around the 0.15% Ni concentration volume. It is the overall association between distributions of mineralization, barren host rocks and seismic texture that present enticing direction for seismic reflection. Textural domaining presents a catalyst for volumetric geological interpretation of 3D seismic reflectivity data. It aids the search for hidden macroscopic geology associations perhaps not previously considered.

Figure 7 provides a larger volumetric representation of seismic texture domains at Kevitsa. Here the “bowl” shape of the rock mass that hosts the Kevitsa deposit is traced out by Cluster I (yellow) and Cluster III (orange). A density log is included on the image and shows a sharp increase in density midway down the hole, associated with a transition from Cluster III (orange) to Cluster I (yellow) dominated rocks.

Seismic textural domaining provides a volumetric summary and a vocabulary for describing seismic texture distribution. While seismic attributes cannot change the information content residing within reflectivity images, they can reveal relationships or patterns hidden within the data. For example, cluster VII (red), in Fig. 6 identifies volumes exhibiting extremes with highest relative localized GLCM contrast texture. The distribution of this anomalous seismic texture can then be isolated, described and volumetrically represented within many cubic kilometers of rock.

Seismic textural domaining is intended to isolate groups or patterns within reflectivity images in the same sense that modern GLCM automated image recognition can be designed to find patterns or objects within videos or photographic images^53,54^.

#### Borehole petrophysics versus seismic textural clusters along the trace of a borehole

Seismic textural domaining provides a statistical representation of the 3D distribution of reflectivity from combinations of dip steered GLCM texture attributes within a seismic volume. The output from this process can be compared with wireline logs along the trace of a drillhole. Before considering comparisons between clusters and logs, it is important to note that; (i) wireline logs provide a representation of petrophysics from tiny volume along the trace of a drillhole (e.g., from a volume much less than a cubic meter), (ii) the drillhole trajectory is rarely uniform or perpendicular to true geological dip along its length. In contrast textural domaining generates a cluster distribution derived, dip steered amplitude independent GLCM textural seismic attributes in a sliding 3D window (see Methods). Despite salient differences in scale and intent of wireline logging versus textural domaining, comparison between the outputs from both is still expected have value in our analysis.

#### Wireline petrophysics versus seismic texture clusters: barren rock

Figure 8 shows wireline logs for velocity, density, total counts gamma and electrical conductivity plotted over a background color representation of textural domains extracted along the trace of Kevitsa drill hole 1. Clusters along the trace of the hole are color coded according to the accompanying Kevitsa ten cluster seismic textural reference diagram. The reference diagram displays the input GLCM texture cluster centroids. The diagram shows the relative contribution of each GLCM seismic texture attribute to the ten cluster centroids.

In Fig. 8, point A identifies shallow textural extremes (e.g., clusters 4 and 5) before a relatively large change in all petrophysical logs, particularly density. In Fig. 8 point B neatly corresponds to (i) a change from lower velocities at shallow depths to higher velocity and (ii) a change from low entropy clusters to high entropy clusters (i.e., dominantly orange and yellow). Point B identifies a major change in seismic texture and the character all wireline logs.

In Fig. 8 point C represents the start of a subtle transition to higher electrical conductivity peaks and is coincident with the subtle textural change from cluster III (orange) to cluster I (yellow). Point C also coincides with a smaller increase in velocity and density (i.e., acoustic impedance). Point D is located at the onset of gradually increasing gamma and velocity towards an abrupt change in gamma and conductivity at point E. It is clear that petrophysical parameters, like gamma and conductivity, need not have a direct link to seismic texture. Despite this, there are fair correlations between the major changes in petrophysical character and distribution of the clusters along the drillhole.

GLCM texture attributes provide information concerning patterns but not absolute amplitude^16^. So, when comparing textural domains to wireline logs its worth also considering the “texture” of the wireline logs. For example, in hole 1 the character of the conductivity distribution between 300 and 500 meters below ground level (mbgl) (i.e., Point C in Fig. 8) is different to that between 500 and 800 mbgl although average conductivity may be similar.

#### Wireline petrophysics versus seismic texture clusters: cluster changes across elevated Ni concentrations

Drillhole 1 intersects barren rock. We now consider drillhole 2 (i.e., Fig. 9) which intersects Ni mineralization. The same set of petrophysical logs (i.e., velocity, density, gamma and conductivity) are included in Fig. 9 along with percent Nickel. Once again, the distribution of Textural clusters is presented as background color behind all logs.

In Drillhole 2, at 450 m depth (i.e., point A), there is a change from Cluster II (blue) to Clusters I (yellow). This transition corresponds to an increase in GLCM entropy and contrast attributes and a decrease in energy and homogeneity attributes. The transition at Point A is also associated with a small but clear increase in velocity and density. In the rock mass above 450 m there are relatively large variations in gamma.

Ni mineralization and electrical conductivity are relatively well matched. However, the zone of elevated Ni and conductivity pay little respect to the change in seismic texture domains at point A. In a highly complex ore body such as at Kevitsa there is no requirement that mineralization, large scale rock texture or indeed seismic texture domains be spatially correlated. For a polymetallic deposit like Kevitsa there were multiple phases of mineralization overprinting mafic layering and different phases of deformation.

Another notable zone from Fig. 8 is between 1100 m and 1150 m. This small petrophysically “dead” zone exhibits little variation in any of the wireline logs and correlates exceedingly well with Textural Cluster VIII (green). Here there is a clear connection between a textural cluster and a distinct lithology identified in the logs.

Analysis of the two drillholes provides some comfort that the gross distribution of clusters does map out zones with common rock mass character. While the mapping is not unique to a rock type, it again provides a new dimension for comprehending and interpreting geology from 3D seismic imaging.

### Example 3: Seismic texture guided cooperative inversion of seismic and magnetotelluric data

Textural domaining of a 3D seismic reflectivity volume is intended as a catalyst to finding hidden geological objects. It may also serve a further function by providing a framework for cooperative inversion intended to significantly improve subsurface imaging with lower-resolution electromagnetic or potential field methods (see Fig. S2). Cooperative inversion includes any beneficial transference of information from one geophysical method to another. The outcome needs to have dependence on inputs from at least two geophysical methods^24,55,56^. Our emphasis is on integration of seismic texture domaining within cooperative inversion strategies.

Seismic waves propagate in the earth with energy reflected and transmitted at interfaces with little net loss of energy from the seismic wavefield itself. In contrast, electromagnetic wave propagation can be highly diffusive and experiences significant losses connected to the earth’s electrical conductivity distribution. This means that while seismic methods can retain resolution to great depths, resolution for electromagnetic methods tends to diminish rapidly with depth. If a link between seismic reflectivity distribution and electrical conductivity distribution can be found, then it should be possible for the seismic information to carry inversion of electromagnetic (EM) data (e.g., magnetotellurics) to a higher resolution that would be not be possible within an independent inversion.

The Nevada 3D seismic reflection data is accompanied by close to 200 full tensor magnetotelluric (MT) measurements on a 3D grid. Survey details, examples of the data acquired and naming conventions are provided in Le. *et al*.^24^. Next, we demonstrate the integration of textural domaining within a strategy for cooperative inversion of seismic and MT data.

For seismic texture guided cooperative inversion, large sub-volumes with common seismic texture (i.e., derived from seven GLCM texture clusters) are recovered from the textural domaining process to provide a prior model framework. Each sub-volume can be assigned a first pass conductivity based on outputs of unconstrained MT inversion or via some form of transfer function.

We computed two standard unconstrained MT inversions. Outcome S1 was generated with a 100 Ω·m halfspace prior model. Outcome S2 was created with a 40 Ω·m halfspace prior model. Le, *et al*.^24^ enlisted the services of classic geometric seismic attributes (e.g., polar dip) in their exploration of cooperative inversion. Here we present a new seismic texture guided cooperative inversion outcomes; C6 and C9. Both these outcomes use a prior model framework generated from textural domaining described above. Outcomes C6 and C9 have the seismic texture guided subsurface framework filled with conductivity values extracted from S2 and S1, respectively. That is, sub-volumes are defined by domains with common seismic texture derived from textural domaining and each sub-volume is populated with a conductivity values that is a statistical average from the MT inversion outcomes S1 and S2 in that sub-volume.

Texture guided cooperative inversion of the Nevada data is run on a Cray XC40 supercomputer system located at the Pawsey Supercomputing Centre; Perth Western Australia. It is encouraging that an exceedingly low RMS error of less than 1.3 % is achieved for output C6. This is a further reduction in RMS error when compared to any strategies detailed in Le. *et al*. 2016 for the same data set. We will extend our analysis of inversion misfit to highlight where the significant improvements in fit between field and final model data after inversion has been achieved for outcome C6 and C9.

**Figure 10:**
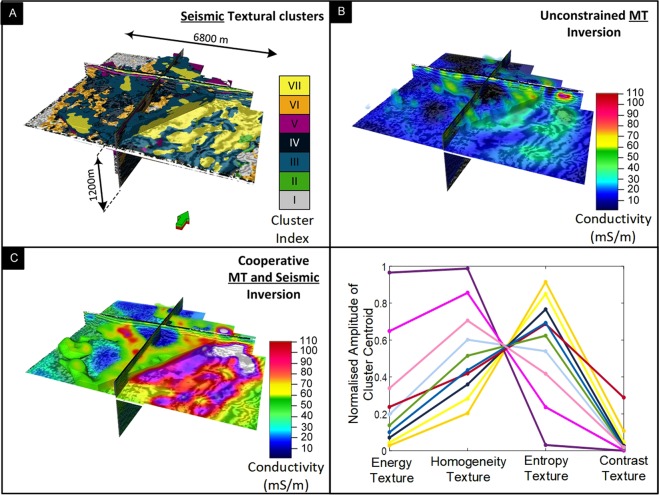
Volume rendered^57^ images of textural domains and conductivity distribution from seismic texture guided cooperative inversion of co-located seismic and MT data from Nevada, USA. The Figure contains (**A**) a 3D representation of seismic texture clusters, (**B**) output geo-electrical distribution from unconstrained MT inversion S1 and (**C**) output geo-electrical distribution from seismic texture guided cooperative inversion. We note that exceedingly low RMS error of 1.3% is achieve for model C6. Cooperative inversion has revealed three channels perpendicular to the major fault.

Figure 10 presents volume rendered images of: (i) seismic textural clusters (ii) conventional unconstrained inversion outcome S1 and (iii) seismic texture guided cooperative inversion outcome C6. The planar cut-surface in Fig. 10 is set at 400 m below ground level and facilitates comparison of the outcome of standard unconstrained inversion S1 with texture guided cooperative inversion C6.

At the shallowest depths, small patches of elevated conductivity tend to form beneath receiver locations in the outcome of unconstrained inversion. This can be referred to as the “receiver footprint”. It is a common artifact in both MT and EM inversion. It occurs when the spatial sampling is insufficient at shallow depths. It is a particular challenge where; (i) data is sparse, (ii) the halfspace resistivity for unconstrained inversion is poorly selected or (iii) the true shallow conductivity distribution is highly complex. After texture guided cooperative inversion there is a significant reduction in receiver footprint because the seismic textural based clusters are able to reasonably define the shallow geological framework. This framework includes shallow channels or high conductivity depression that can be populated with reasonable initial conductivity values.

The detailed conductivity distribution revealed after seismic texture guided cooperative inversion (C6 and C9) elevates the value of the MT survey^24^. The geometry of the major fault, a set of shallow channels and cover thickness are clearly revealed in the conductivity distribution derived from seismic texture guided cooperative inversion.

For example, the geometry of seismic texture Cluster VII (yellow) is sufficient to guide the MT inversion outcome towards detailed shallow three-dimensional relatively high conductivity channel features incised in basement. These run south-east towards the major fault. While there are hints of these features in the unconstrained MT inversion outcome, they are not readily interpretable. Next, we examine of cooperative inversion outcomes along the trace of Nevada drillhole 1 and then consider detailed 3D misfit distribution for inversion outcomes S1, S2, C6 and C9.

**Figure 11:**
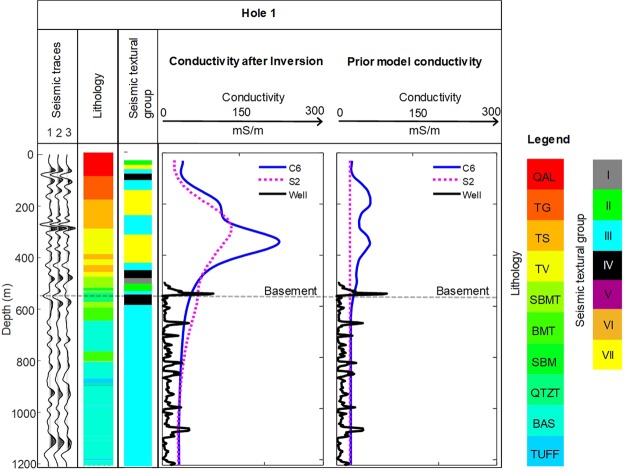
Comparison of seismic traces, lithology, conductivity from drillholes logs, conventional unconstrained MT inversion and texture guided cooperative inversion outcomes, along the trace of drill hole 1. The prior model conductivity for unconstrained and texture guided cooperative inversion is shown in the right hand panel. Immediately to the left of curves for the prior model we present the final unconstrained and texture guided cooperative inversion outcomes. We observe that the texture guided cooperative inversion, which is run on a Cray Cascade XC40 Super Computer, has significantly changed the conductivity range but retained the general geometry of cover and basement inherited from the Seismic Texture distribution. The final cooperative inversion RMS misfit of 1.3. This is exceedingly small. Lithology abbreviations are: QAL Tertiary volcaniclastics, tuffs and siltstones. TG Rhyodacite volcanics Tuffaceous silt and clay. TS Tuffaceous silt and clay. TV Green glassy volcanics. SBMT Siliceous mudstone with tuff. BMT Mudstone with tuff. QTZT Quartzite. BAS Basalt. TUFF Tuff. Note that Fig. 11 is presented in a similar format to that shown in Le, *et al*.^24^ so that comparison to alternative inversion strategies can be made.

Figure 11 below compares (i) seismic traces, (ii) lithology (iii) an electrical wire line log, (iv) unconstrained MT inversion outcome S2, and (v) seismic texture guided cooperative inversion outcome, C6, along the trace of drillhole 1. The right hand panel shows prior model conductivity for inversion strategy C6 and S2. The panel immediately to its left shows a comparison of the outcome of unconstrained inversion and our texture guided cooperative inversion. There are significant differences between unconstrained and cooperative inversion outcomes. Although smoothed, the large changes in conductivity from cooperative inversion outcome C6 conform reasonably to the lithological groupings. Basement conductivity on average appears to be slightly higher for outcome C6 than is indicated by the wireline logs, although it’s worth noting that basement is electrically heterogeneous with small intervals (less than 10 m) of relatively elevated conductivity. The cooperative inversion strategy used to generate outcome C6 deployed a uniform smoothness constraint throughout the model domain. That is, a constant covariance coefficient of 0.25 was selected for the whole model^24^. Figure 11 provides a highly local but important comparison of the inversion outcomes with a drill hole within an MT derived conductivity volume that spans more than 100 cubic kilometers.

#### Seismic texture guided cooperative inversion; analysis of “3D misfit” distribution

**Figure 12:**
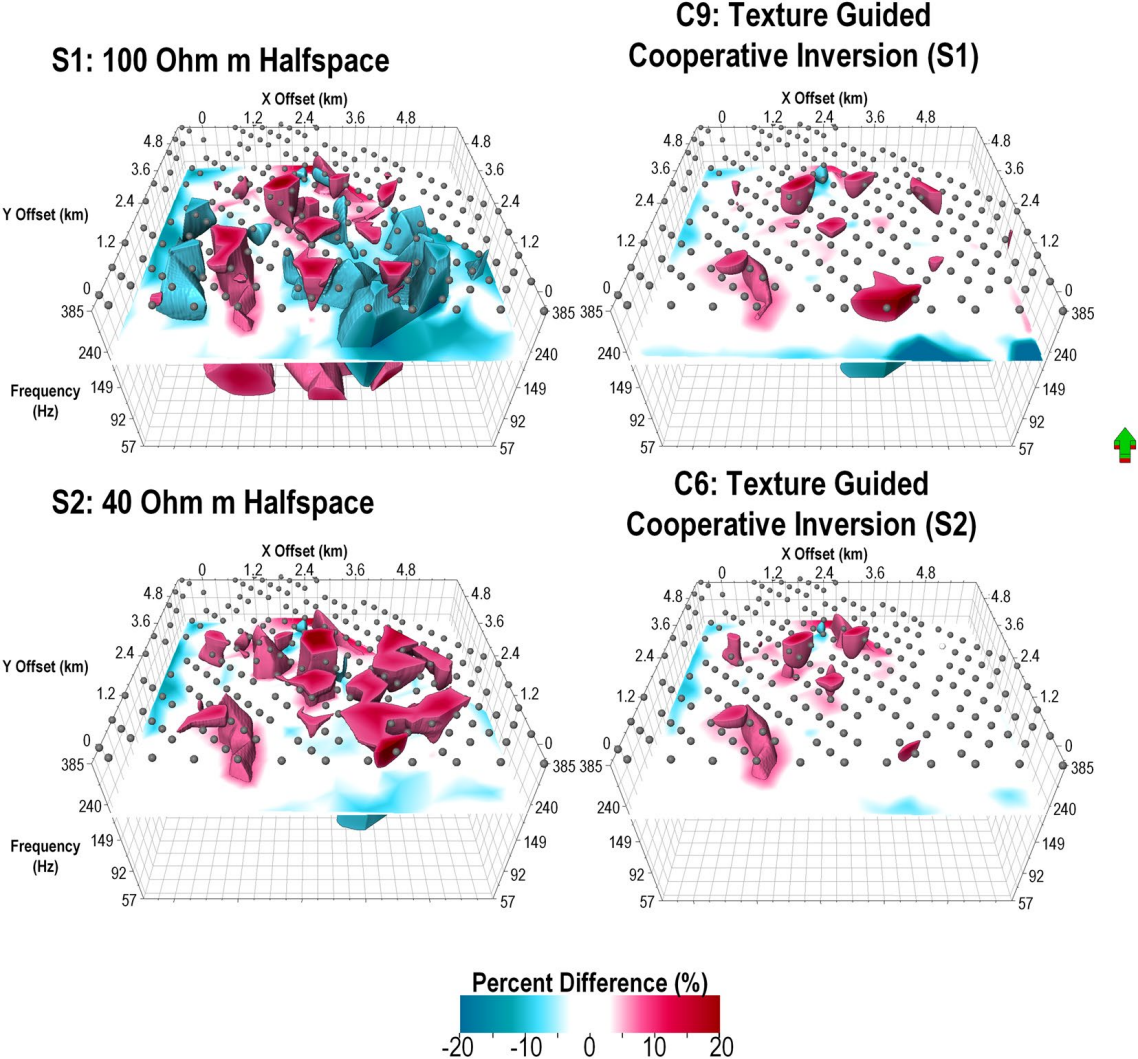
Volume rendered^57^ 3D misfit images from unconstrained and cooperative inversion of MT data at Nevada. A 3D representation of data misfit for unconstrained inversion outcome S1 and S2 can be compared misfit after texture guided cooperative inversion C6 and C9. The small gray spheres denote the locations of each surface MT measurement. The vertical Z axis is frequency in Hertz. The colors represent the raw percent difference between model and field data after the final inversion. The extremes in percent difference are rendered blue (negative) and red (positive) and a frequency slice is shown at about 200 Hz. The image highlights the substantial reduction in misfit for the cooperative inversion outcomes at each station. In particular the cooperative inversion outcome, C6, presents with significant improvement— approaching zero percent misfit across the major fault and over the thick cover region to the right on the 3D images (see Figs 4 and 5).

Figure 12 includes a 3D representation of percent misfit versus frequency (z-axis) below each MT station for inversion outcomes S1, S2, C6 and C9. In Fig. 12, misfit is defined as the residual error between the synthetic and real data divided by the absolute (abs) value of the diagonal transverse magnetic (TM) component (Z_yx_). This is expressed in the equations below.

1 $${\epsilon }=\frac{|{Z}_{syx}|-|{Z}_{dyx}|}{|{Z}_{dyx}|}$$

Here ${\epsilon }$ is the residual error (expressed as percentage in Fig. 12), ${Z}_{syx}\,\,$is modeled (i.e. calculated) TM Impedance and ${Z}_{dyx}$ is field (i.e. measured) TM Impedance.

We highlight the significant improvement in the quality of the fit between field and model data (after inversion) for texture guided cooperative inversion outcomes C6 and C9. The improvements are located in the critical zones across the major fault; a key sub-vertical geo-electrical and acoustic interface. That is, in the fault zone there is a large reduction in percent error for inversion outcomes, C6 and C9. Achieving these excellent fits at higher frequencies may be critical. Poor fits at higher frequencies often link to incorrect shallow conductivity distributions. These can translate to incorrect conductivity distribution at greater depths.

As noted earlier, a challenge for MT inversion is the “receiver foot-print”. This can occur where the inversion attempts to fit the field data by creating artificial localized zones of anomalous conductivity below each MT receiver station. Creation of a geometrically realistic prior conductivity distribution at shallow depths based on 3D seismic texture alleviates these problems. In Fig. 12 we observe the exceedingly small misfit for frequency up to 400 Hz for the vast majority of stations for texture guided cooperative inversion strategies C9 and C6. Inversion outcome C6 shows a decrease in misfit proximal to the down thrown side of the major fault where the high conductivity cover thickens to over 500 m.

Next we use volume rendering to examine the detailed conductivity distribution achieved by strategy C6. These are compared to various representations of the 3D seismic reflectivity data.

#### Volume rendering cooperative inversion outcome C6 with reference to seismic data

**Figure 13:**
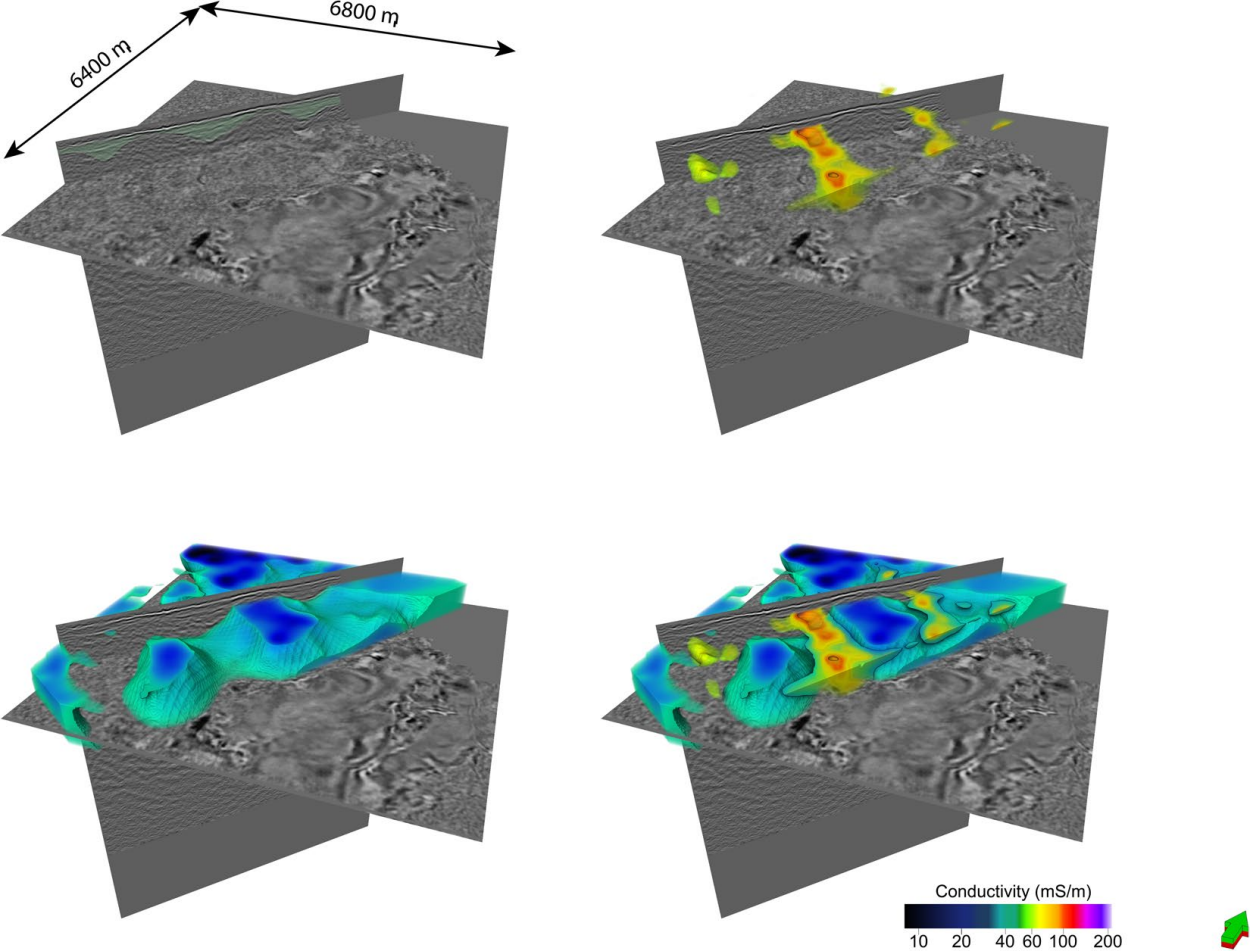
Volume rendered images showing high conductivity channel geometries resolved by seismic texture guided cooperative inversion strategy C6. The channels exhibit relatively high GCLM homogeneity and low entropy. The seismic textural domaining has provided a shallow detailed framework that is integrated into the texture guided cooperative inversion. The high conductivity channels or depressions that appear to be incised in basement become well defined by texture guided cooperative inversion strategy C6.

**Figure 14:**
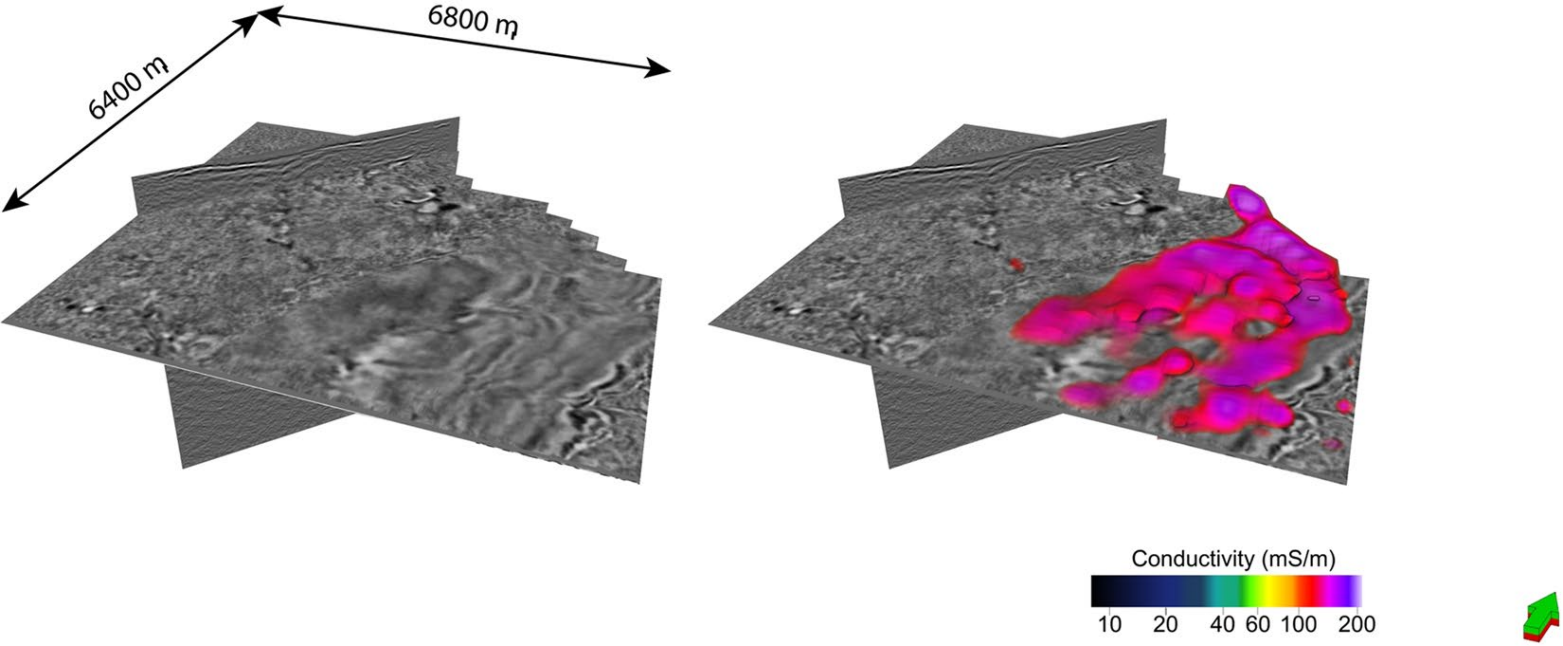
Volume rendering of the highest electrical conductivity formations within cover sequences. Conductivity is derived from texture guided cooperative inversion strategy C6. Our 3D inversion misfit analysis shows that strategy C6 generates exceedingly good fit between field and model MT data for the thick cover sequences (see Fig. 12).

**Figure 15:**
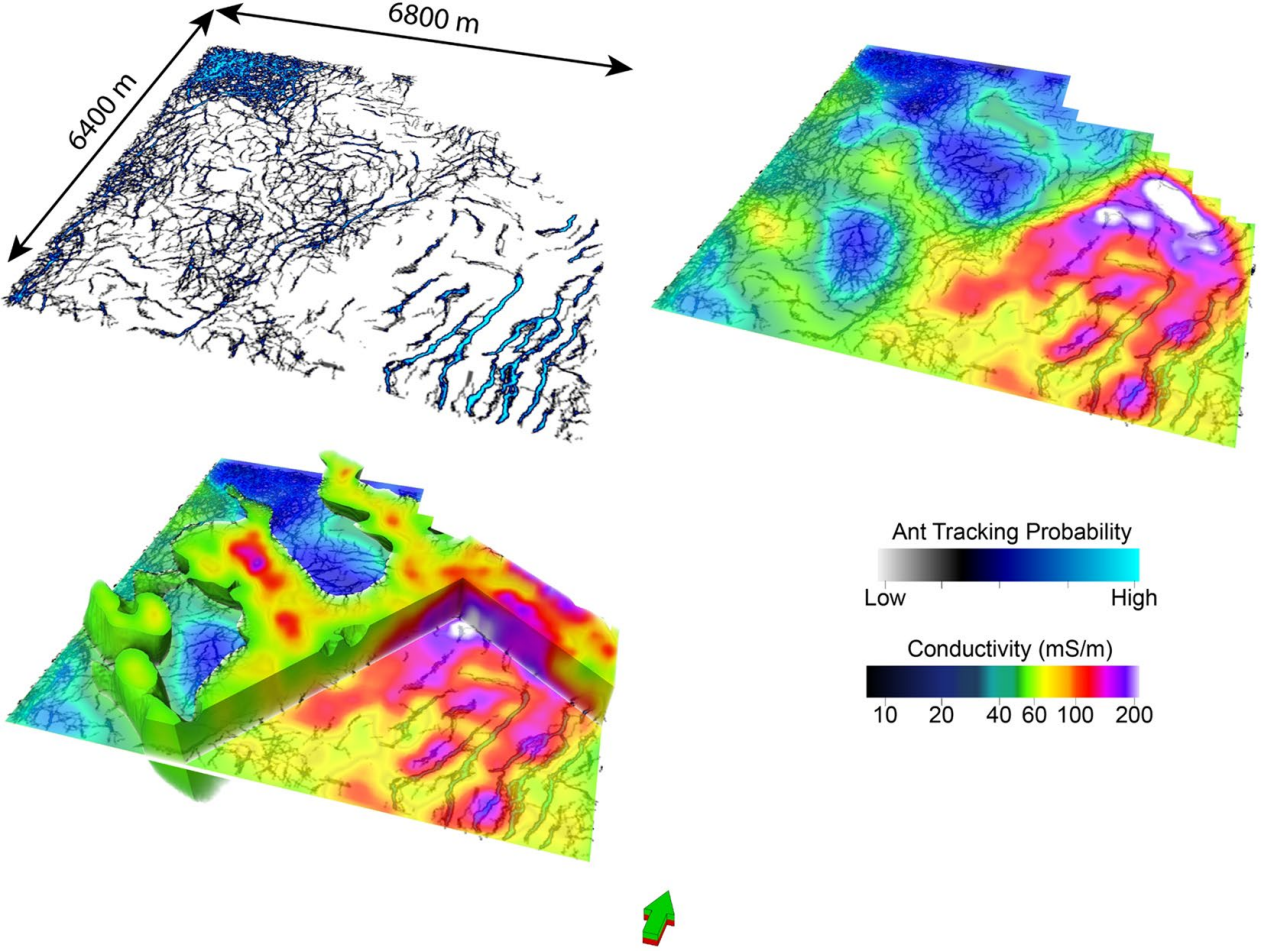
Images comparing ant tracking probability^57^ within the seismic reflectivity volume with the outcome from seismic texture guided MT inversion strategy C6. Note how well the electrical conductivity distribution maps to the orientation, concentration and character of ant tracks.

We provide three volumetric representations^57^ of conductivity distribution resulting from inversion strategy C6 as Figs 13–15. In particular we consider; (i) shallow channels leading towards the down through fault block, (ii) distribution of highly conductive sediments within cover of the down thrown fault block and (iii) a comparison of the distribution of conductivity with the outcome of an ant tracking algorithms applied to the seismic volume^51,57^:

1. Figure 13 shows rendering of three high electrical conductivity channels like structures set within higher resistivity basement rock. The four images show (i) seismic with channel like features marked in green, (ii) the high conductivity zone, (iii) geometry of higher resistivity rock and (iv) rendering of both high and low conductivity zones. These channels like structures appear to be incised in basement. They run towards and over the edge of the major fault. The framework of the structures is broadly defined by seismic texture cluster I (yellow). Cluster I is characterized by relatively high GLCM homogeneity and low GLCM entropy compared to surrounding rock. That is, the frameworks is established by the textural domaining and the detail of the conductivity distribution is etched out by the texture guided cooperative inversion, with the assistance of a Cray Cascade supercomputer.
2. Figure 14 shows the distribution of high conductivity sediments within younger thick cover sequences on the down thrown side of the major fault. Texture guided cooperative inversion strategy C6 has been able to refine conductivity distribution within the cover sediments such that the geometry of the conductive zones follows the geometry of shallow dipping sequences within the seismic reflectivity volume.
3. Figure 15 compares ant track probability^51,57^ with electrical conductivity from texture guided Cooperative Inversion outcome C6. The higher conductivity zones tend to correlate with absence or low probability of ant tracks, while areas with a high density of short randomly oriented ant tracks is correlated with highest electrical resistivity. For example, the intense chaotic high probability ant tracks at the rear of the image, map to a 3D volume of high electrical resistivity. This zone is also neatly defined by cluster I (gray) characterized by the highest GLCM entropy and the lowest GLCM homogeneity centroids as show in the Nevada seven cluster seismic texture reference diagram. Again, seismic texture distribution is able to guide the cooperative inversion to a new outcome that characterizes a rock volume by multiple parameters. The ant tracking algorithm provide an alternative independent representation of a textural aspect of the 3D seismic reflectivity imaging.

In summary, integrated strategies such as texture guided cooperative inversion can provide insight into the subsurface that no single method can hope to deliver.

## Discussion

Subtle changes in seismic texture cannot be systematically mapped by the human interpreter within 3D seismic reflectivity imaging that may span tens or even hundreds of cubic kilometers. Multivariate statistical clustering or similar methods are needed. We have presented pathways for partitioning a reflectivity volume into discrete textural domains. Under ideal circumstances, our seismic texture domaining (i.e., the clusters) will link with rock mass that has mappable geological and or geotechnical character.

Modern seismic reflection methods are rapidly converging to consistent high quality true relatively amplitude processing^2^. However, it should be recognized that recovery of 3D seismic reflectivity from raw field records (e.g., pre-stack shot records) may not always generate a precise representation of true subsurface relative reflectivity distribution. In this case it is possible for some clusters to map to artefacts or inadequacies inherited from seismic processing or acquisition. Further we acknowledge the importance of choosing a meaningful number of clusters for K-means analysis. If a large number of clusters is chosen to represent a seismic reflectivity volume, then it is likely that sets of clusters will be texturally gradational and, in this case, it may be difficult for any single cluster to map the boundaries of a distinct rock mass. Certainly, internal clustering validation measures provide the baseline quantitative method to determine the natural or optimal number of clusters for K-means analysis. However, the objective of the clustering will also be a driver for selecting cluster numbers. In practice there is considerable benefit, (i.e., for interpretation of geology), combined with negligible additional effort required to compute textural domaining over a range of cluster numbers (see Fig. 3).

The “best” circumstance for mapping seismic texture domains to a distinct rock mass is when the nature of reflectivity persists and repeats throughout a volume of rock. For example, a persistent chaotic, high-contrast reflectivity domain will be readily mapped against a chaotic but low-contrast domain by seismic textural domains in circumstances where it’s highly improbably that this type of subtle but quantifiable change in seismic texture can be mapped by an interpreter.

An opposite and unlikely textural extreme would exist if all rock volumes were uniform (or varied slowly) in velocity and density. In this case the cluster representing each volume must represent the absence of reflectivity. In this end member scenario, where large rock volumes are uniform, textural domaining can only map out differences in seismic reflectivity along dividing boundaries. Fortunately, perfectly uniform rock volumes are exceedingly rare in nature.

Textural domaining provides one representation of the seismic reflective data and it’s necessary to reference interpretation to the original reflectivity imaging. It may be also helpful to map textural domains over other seismic attributes like cosine of phase. In Figs 6–9, this was achieved directly by superimposing the cosine of phase attribute onto the textural clusters. Here, we directly observe the geometry of reflections relative to clusters and can intuitively assess how K-means analysis has statistically partitioned textually similar domains within the seismic volume. Visual inspection of clustering outcome versus original seismic imaging is necessary and for the examples, it provided considerable comfort that textural domaining had indeed identified significant changes in seismic texture (i.e., see Figs 7 and 9).

Ultra-high resolution seismic^58,59^ and deep learning^43^ algorithms are on our doorstep and processes like those represented in Fig. 2 will likely drive new outcomes that can be automated by high-level code^34,40,60^. In the near future, it should be possible to apply processes like seismic textural domaining across multiple 3D seismic surveys to trace geologically environments or even mineralization throughout entire geological provinces.

## Conclusion

Seismic reflection methods are often essential for subsurface exploration (e.g., hydrocarbon, mineral, water, geothermal) and earth science research. We have provided new techniques and examples for extracting geological meaning from reflectivity data based on seismic texture. A set of gray-level co-occurrence matrix textural seismic attributes is combined with K-means analysis for classifying the 3D seismic volume into seismic domains with common texture. Images of textural domains are accompanied by a seismic texture reference diagram. Selection of the optimum number of attributes is assisted by internal clustering validation measures. The combination of seismic textural domaining with the seismic texture reference diagram is unique and can reveal subtle variations in subsurface rock mass character. Under some circumstances the seismic texture clusters may directly recover the volumetric distribution of geologically similar rock mass.

The method is tested on high-resolution 3D land seismic reflectivity data sets from Nevada, USA and Kevitsa, Finland. We have chosen examples from hard-rock terrains where seismic reflection data is poorly endowed with continuous distinct reflections. The value of our methods becomes clear in these challenging terrains where exceedingly subtle variations in acoustic impedance distribution can be revealed as texturally distinct domains. For the Nevada example, we are able to highlight shallow channels and zones within fault transitions that are texturally distinct. We also demonstrate how textural domaining may feed into and improve cooperative inversion. For the example from Finland we show relationships between Ni Concentration at the polymetallic Kevitsa mine site and distribution of seismic texture domains.

Textural domaining provides significant steps towards mapping volumetric distributions of seismic reflectivity linked with macroscopic geological environments and processes. Seismic texture domaining and accompanying reference diagrams provide insight into the subsurface and will hopefully act as a catalyst for related methods of extracting geological meaning from increasingly cost-effective, information rich, high-resolution 3D seismic reflectivity images.

We have presented pathways to unsupervised learning to find hidden geological objects based on dip steered GLCM seismic textures and we have used the outcome within cooperative inversion of seismic and magnetotelluric data to generate a significantly improved conductivity volume.

## Methods

We have developed a method for domaining any seismic reflectivity volume based on seismic texture. A set of gray-level co-occurrence matrix (GLCM) textural seismic attributes^16–19^ is combined with K-means analysis^21–27^ for classifying any 3D seismic volume into domains with common texture.

New volume rendered images of textural domains are accompanied by a seismic texture reference diagram. The seismic texture reference diagram provides the set of mean values of the seismic texture attributes and these mean values characterize each cluster. Put simply, the reference diagram provides the textural character for each cluster or domain. Our first example from Nevada provided the seven-cluster texture reference diagram with the means of the four GLCM seismic texture attributes for Nevada reflectivity volume shown in Fig. 3. Selection of the optimum number of attributes is also assisted by internal clustering validation measures^47,61^.

The method requires a sequence of steps culminating in the generation of (i) a 3D volume that is composed of a finite set of domains with common seismic textures, and (ii) an accompanying seismic texture reference diagram. By textural “domaining” we mean the process of subdividing the reflectivity volume into a number of domains that are texturally similar. Core steps for seismic texture domaining are:

(i) Computation of a “dip-steering” cube from the seismic reflectivity volume^50,62,63^,
(ii) Conversion of seismic reflectivity data to a gray-level co-occurrence matrix (GLCM)^17,64^,
(iii) Computation of textural attributes from the GLCM^16–19^,
(iv) K-means cluster analysis^21–27^ to recover domains with common seismic texture,
(v) Computation of internal clustering validation measures,
(vi) Computation of the seismic texture reference diagram, and
(vii) Volume rendering and comparison of seismic texture, seismic reflectivity and textural clusters.

Below we detail each step, working from dip steering, to computational requirements for GLCM textural attributes and K-means cluster analysis.

### Dip steering, textural seismic attributes, and cluster analysis

The four textural attributes, energy texture, entropy, homogeneity, and contrast^16–19^ are used as input to K-means clustering. The textural attributes are computed in a small 3D sliding calculation window that glides through the full seismic volume.

We have applied “dip steering” for calculation of textural attributes within the OpendTect software^50,62,63^. The dip-steering cube is used to ensure that the attribute calculation window follows the seismic dip. For dip steering, seismic dip and azimuth are calculated at every sample position based on analysis of reflectivity in a sliding 3D window. Dip steering enhances the quality of attributes or picking horizons^50,65,66^.

Seismic amplitudes in the 3D sliding calculation window can be considered as gray-scale levels or gray levels of pixels (i.e., pixel intensity). Such data can be converted to a 2D GLCM reflecting the relative frequency of concurrence of pairs of pixels with specific values in a specified spatial relationship within the 3D calculation window^16,50,67^.

Each of the seismic textural attributes is calculated from the GLCM^17,64^. The GLCM provides a statistical characterization of seismic data within the calculation window. Once the GLCM is computed for the sliding 3D calculation window^16,50,64^ the attributes, energy texture, entropy, contrast and homogeneity, are computed. The mathematical expression for each textural attribute are written in the work, Seismic attributes for prospect identification and reservoir characteriztion^16^ and are provided below:

2 $${\rm{Energy}}=\sqrt{\mathop{\sum }\limits_{{\rm{i}},{\rm{j}}=0}^{{\rm{N}}-1}{{\rm{P}}}_{{\rm{i}},{\rm{j}}}^{2}}$$

3 $${\rm{Entropy}}=\mathop{\sum }\limits_{{\rm{i}},{\rm{j}}=0}^{{\rm{N}}-1}{{\rm{P}}}_{{\rm{i}},{\rm{j}}}(-\mathrm{ln}({{\rm{P}}}_{{\rm{i}},{\rm{j}}}))$$

4 $${\rm{Contrast}}=\mathop{\sum }\limits_{{\rm{i}},{\rm{j}}=0}^{{\rm{N}}-1}{{\rm{P}}}_{{\rm{i}},{\rm{j}}}{({\rm{i}}-{\rm{j}})}^{2}$$

5 $${\rm{Homogeneity}}=\mathop{\sum }\limits_{{\rm{i}},{\rm{j}}=0}^{{\rm{N}}-1}\,\frac{{{\rm{P}}}_{{\rm{i}},{\rm{j}}}}{1+{({\rm{i}}-{\rm{j}})}^{2}}$$

Where ${{\rm{P}}}_{{\rm{i}},{\rm{j}}}$ is the i^th^ row and j^th^ column of the GLCM matrix P.

Each textural attribute is sensitive to a different aspect of seismic texture. The energy texture seismic attribute highlights zones of high textural stability within each calculation window. It is independent of amplitude and is suited to highlighting continuity. Energy texture should not be confused with any of the standard energy attributes. Entropy is a measure of the level of randomness in the calculation window. Homogeneity expresses a type of smoothness in reflectivity and contrast emphasizes differences in amplitude.

Yenugu, *et al*.^68^ systematically reviewed textural attributes, tracing the history of Gray-Level Co-occurrence Matrix^17^ to modern applications in seismic reflectivity, especially for hydrocarbon reservoir characterisation^16,18,19^.

A 3D sliding calculation window is needed to compute 3D seismic attributes. In the time domain, the window consists of three parameters: (i) the time gate, (ii) the inline step out, and (iii) the crossline step out. In the depth domain, the time gate is replaced by a depth gate. In our examples, the depth gate will be set to approximately 16 m and the step outs for inline and crossline traces close to 40 m, which includes about 25 traces in the sliding calculation window. We systematically tested larger calculation windows for computing textural attributes but found the loss in detail rapidly became unacceptable, which is entirely consistent with statements made by Chopra, S. and Marfurt, K. J.^16^ in their book on seismic attributes.

After computation of the four dip-steered textural attributes, the next action is to partition or domain the full seismic volume into domains with statistically similar seismic texture. This was done by cluster analysis. A cluster or class can be described by a set of mean values of the input seismic attributes. Each cluster represents one of a finite set of characteristic seismic textures. In our approach the four seismic texture attributes, energy, homogeneity, entropy and contrast are scaled from 0 to 1 as suggested by Samarasinghe, S.^69^ according to:

6 $${{\rm{a}}}_{{\rm{newi}}}=\frac{{{\rm{a}}}_{{\rm{i}}}-{{\rm{a}}}_{{\rm{\min }}}}{{{\rm{a}}}_{{\rm{\max }}}-{{\rm{a}}}_{{\rm{\min }}}}$$

Where ${{\rm{a}}}_{{\rm{i}}}$ is an observation of an attribute value a. Here ${{\rm{a}}}_{{\rm{\min }}}$ and ${{\rm{a}}}_{{\rm{\max }}}$ are minimum and maximum values of the attribute a, respectively and a_newi_ is the scaled value of a_i_.

The K-means algorithm is a powerful clustering technique. It is well-tested and readily implemented for many geophysical research applications^21–27^. Lindsten, *et al*.^70^ noted that the core idea behind the K-means clustering technique was first suggested by Hugo Steinhaus in 1957. The approach “minimises the sum, over all clusters, of the within-cluster sums of point-to-cluster-centroid distances”^71^. The method is defined in the equations below which are reproduced from Shen, J., Chang, S. I., Lee, E. S., Deng, Y. and Brown, S. J. (2005), and Lindsten, F., Ohlsson, H. and Ljung, L.^26,70^:

7 $${\rm{E}}=\mathop{\sum }\limits_{{\rm{i}}=1}^{{\rm{k}}}\mathop{\sum }\limits_{{\rm{j}}=1}^{{{\rm{n}}}_{{\rm{i}}}}{\rm{d}}({{\rm{x}}}_{{\rm{ij}}},\,{{\rm{m}}}_{{\rm{i}}})$$

8 $${\rm{d}}({{\rm{x}}}_{{\rm{ij}}},\,{{\rm{m}}}_{{\rm{i}}})=({{\rm{x}}}_{{\rm{ij}}}-{{\rm{m}}}_{{\rm{i}}}){({{\rm{x}}}_{{\rm{ij}}}-{{\rm{m}}}_{{\rm{i}}})}^{{\rm{T}}}$$

Here E is a cost function^26^ and is the sum of square-errors for all observations in the data; ${{\rm{x}}}_{{\rm{ij}}}$ is the jth observation in the ith cluster and ${{\rm{m}}}_{{\rm{i}}}$ is the center value or mean of the cluster. The value i, within ${{\rm{n}}}_{{\rm{i}}}$ is the total number of observations in each cluster i, and k is the number of clusters. Note that the number of clusters should be defined a priori. Minimization of the cost function E can produce k cluster centroids from the space, which are formed by all the observations (x). A summary of the steps needed for the K-means approach are:

**Step 1:** Select the number of clusters (user defined).

**Step 2:** Randomly place *k* points into the volume of the seismic attributes that are clustered. These *k* points express initial cluster centroids.

**Step 3:** Allocate each observation to the cluster that has the closest centroid.

**Step 4:** When each cluster has its observations from its initial cluster centroid, redefine the positions of the cluster centroid by calculating a mean value of its observations. Then, continue to recompute other cluster centroids.

**Step 5:** When *k* new cluster centroids are calculated, repeat Step 3 and Step 4 until the centroids do not change anymore.

The selection of the number of clusters for k-means algorithm is considered for the success of seismic interpretation in building seismic texture domains. We have employed two algorithms, as implemented in the MATLAB code, *evalclusters*.*m*, to evaluate the optimum number of clusters. These are based on high intra-cluster and low inter-cluster similarity and are also known as internal clustering validation measures^61^.

Specifically, we have employed the Davies-Bouldin and Calinski-Harabsz validation measures^47^. The Calinski-Harabasz criterion evaluates the cluster quality based on the ratio of values between and within cluster sums of variance squares^61^. The larger the ratio the better the clusters are defined.

On the other hand, the Davies-Bouldin criterion is based on the ratio of within-cluster and between-cluster distances with the “best” clustering solution having a small Davis-Bouldin index. Since this criterion is a ratio between coherency within clusters and distance between clusters^47^, this number tends to be minimized with relatively few clusters.

Calculating these criteria for high numbers of clusters can become computationally intensive. For the Nevada field example, it took several days to return a value with a standard core I7 Intel ® Xeon ® CPU E5-1650 at speed 3.2 GHz for 11 K-means clusters using the four textural seismic attributes derived from the Nevada reflectivity volume. Certainly, modern supercomputing can make short work of such problems and indeed we use a high-end petascale Cray XC40 supercomputer system for integrating textural domains into cooperative inversion (see Supplementary Information: S2. MT Inversion and Cooperative Inversion).

## Supplementary information

Supplementary Information

## Data Availability

Methods required to compute textural attributes are supplied in this manuscript. The 484 kilometer long Yilgarn Craton - Officer Basin - Musgrave Province (YOM) regional 2D seismic transect is freely available from Geoscience Australia. The Nevada data are supplied and owned by Barrick Gold Corporation. Restrictions apply to the availability of these data, which were used under license for the current study. The Kevitsa data has previously been made available through the Frank Arnott Award. Request for access should be made via www.frankarnottaward.com or directed to Boliden Kevitsa. The Authors are happy to provide contacts within Barrick Gold or Boliden if required.
