# Supplementary material for: New perspectives on Solid Earth Geology from Seismic Texture to Cooperative Inversion
Source: Sci Rep. 2019 Oct 14;9:14737. doi: 10.1038/s41598-019-50109-z (PMC6791834; doi:10.1038/s41598-019-50109-z)
Supplement: Supplementary file 1 — Supplementary Information [file 41598_2019_50109_MOESM1_ESM.docx]

#

# Supplementary Information: New perspectives on Solid Earth Geology from Seismic Texture to Cooperative Inversion:

# Cuong V. A. Le^1,2,3^, Brett D. Harris^*1,2^ and Andrew M. Pethick^1,2^

^1^Curtin University, Western Australian School of Mines: Minerals, Energy and Chemical Engineering, Australia

^2^Deep Exploration Technologies Cooperative Research Centre (DET CRC), Perth, Australia

^3^Ho Chi Minh City University of Science, Vietnam National University

e-mail: B.Harris@curtin.edu.au

+these authors contributed equally to this work

# S1. Sites:

Seismic texture domaining has been completed on high-resolution seismic data sets from Nevada USA and Kevitsa, Finland. The first example has been provided by Barrick Gold Corporation and uses a high-resolution seismic data set spanning more 40 square kilometers. The data set is acquired in the famous Carlin-style gold district of Nevada USA. The second example, from Finland, includes a 3D high-resolution seismic reflectivity data set collected over the active Kevitsa polymetallic mine^1-3^. The locations of both sites are provided in Figure 2.

# S1.1. Nevada, USA

The Nevada site is characterized by thick highly variable cover sequences that range from less than 100 m to more than 500 m below surface across a major fault. The Nevada site and data are fully described in the published Journal papers: (i) Cooperative Joint Inversion of 3D Seismic and Magnetotelluric Data: With Application in a Mineral Province^4^, and (ii) Semiautomatic and Automatic Cooperative Inversion of Seismic and Magnetotelluric data^5^. A first-order challenge at the Nevada site is characterizing and differentiating thick cover sequences from geologically older prospective basement rock. Hillis et al.^6,7^ emphasizes the importance of developing new technologies suited to exploring for Tier 1 mineral deposits under deep barren cover and the Nevada example is particularly salient in this respect.

Data quality for the Nevada 3D seismic is high with inline and crossline spacings of about 17 m and a depth sampling for seismic traces of about 0.61 m. The full reflectivity image consists of 378 inlines × 410 crosslines (155,769 traces with a total of 467,462,769 samples). The Nevada 3D seismic survey is also spanned by a 3D tensor MT survey making it an ideal test site for cooperative inversion.

# S1.2. Kevitsa, Finland

The Kevitsa seismic survey in Finland was completed in a hard-rock environment over, what is now, an active mine. The geology from Kevitsa in Finland is strongly 3D and in places the polymetallic ore zone is crosscut by prevailing seismic dip. The Kevitsa deposit is expected to contain 240 million tons^2^ (using a nickel cut-off grade of 0.1%). The hosting intrusion varies from gabbro to dunite composition, with distinct magmatic pulses likely to be responsible for these different phases. The mineralized zone resides in what is dominantly olivine pyroxenite^2^. The main mineralization, which relates to the disseminated sulfide Ni-Cu-PGE deposit, is believed to be both lithologically and to some extent structurally controlled^3^. Sub-horizontal seismic reflectors throughout the resource area have previously been ascribed to internal layering marked by changes in composition resulting from the distinct magmatic pulses^2^. An interesting aspect of this site is the modest to high electrical resistivity mineralized zone (i.e., Nickel, Copper and Platinum Group Elements) set within the high-resistivity Pyroxenite host rock^1,8^. Figure S1 provides a volume 3D rendered image of the Kevitsa seismic texture Cluster III (orange) and the main 0.15 Ni shell. Note that in this view Cluster III appears to wrap around the mineralized zone.


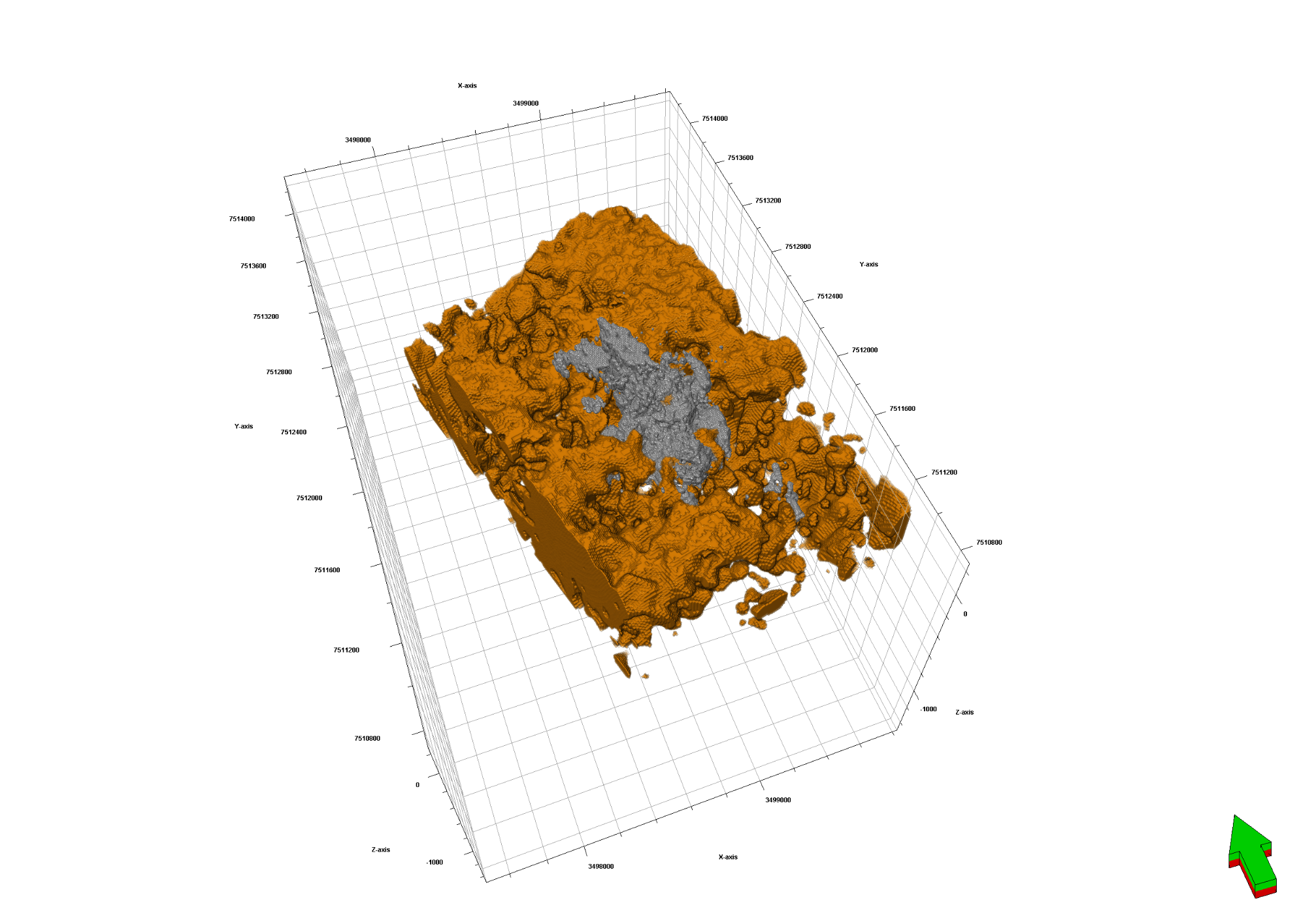


**3400 m**

**240 m**

**1240 m**

**240 m**

**1240 m**

**Z-axis**

**Z-axis**

**North**

**2300 m**


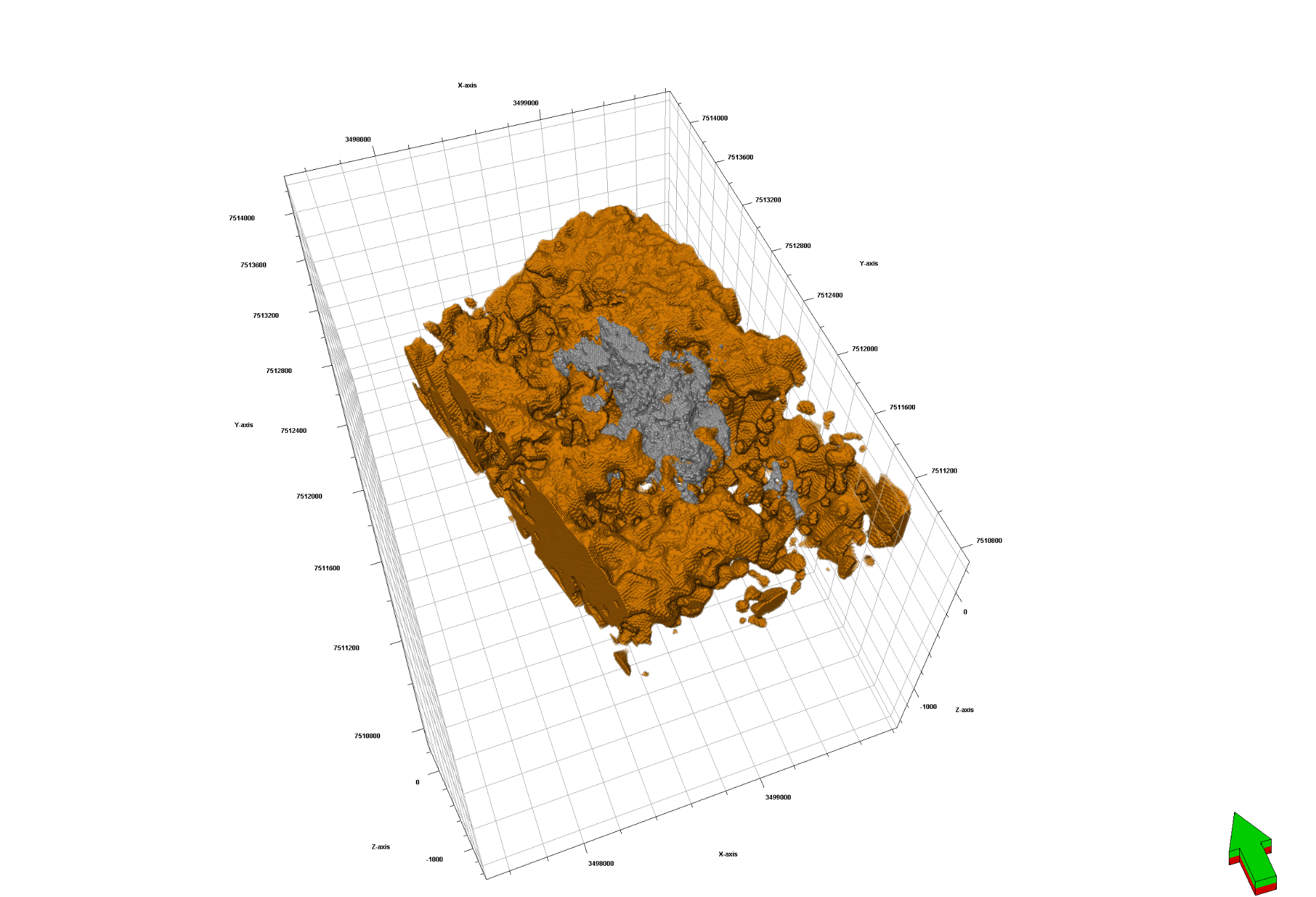


**Depth at 70 m**

**Figure S1.** A 3D volume rendering the Kevitsa Seismic Texture Cluster III (orange) and the 0.15 % Ni concentration shell (metallic gray). Cluster III appears to wrap around the mineralization with little overlap into the high nickel concentration shell.

# S2. MT inversion and Cooperative inversion

MT Inversion of field measurements to subsurface conductivity distribution requires iteratively adjusting a numerical model of subsurface conductivity distribution in a way that decreases the residual difference between the field and modelled data ^9,10^. For MT inversion we have used the ModEM3D code^10,11^ within the Cooperative Inversion framework outlined in Le, et al. ^5^.

Cooperative inversion is the beneficial transference of information from one geophysical technique to another to improve the recovery of subsurface rock properties^12^. The new seismic texture domains may provide volumetric links to macroscopic rock volumes with characteristic electrical properties and so may can be well suited to cooperative inversion.

A flow schematic of processes needed for seismic texture domaining and cooperative inversion of magnetotelluric and seismic data is provided in Figure S2. The technique for creating the prior conductivity model is called “geometric mapping”^5^. It uses the distribution of a seismic texture domains to divide the complete seismic volume into smaller subvolumes. Each subvolume is subsequently allocated on electrical resistivity, based on analysis of distribution of resistivity extracted from unconstrained inversion of the halfspace prior model at 100 Ω·m ^5^. Once each seismic domain is statistically assigned a conductivity, this conductivity distribution feeds a final 3D MT inversion, which is run with the ModEM3D code run on a petascale Cray XC40 supercomputer (i.e., see Figure 10).


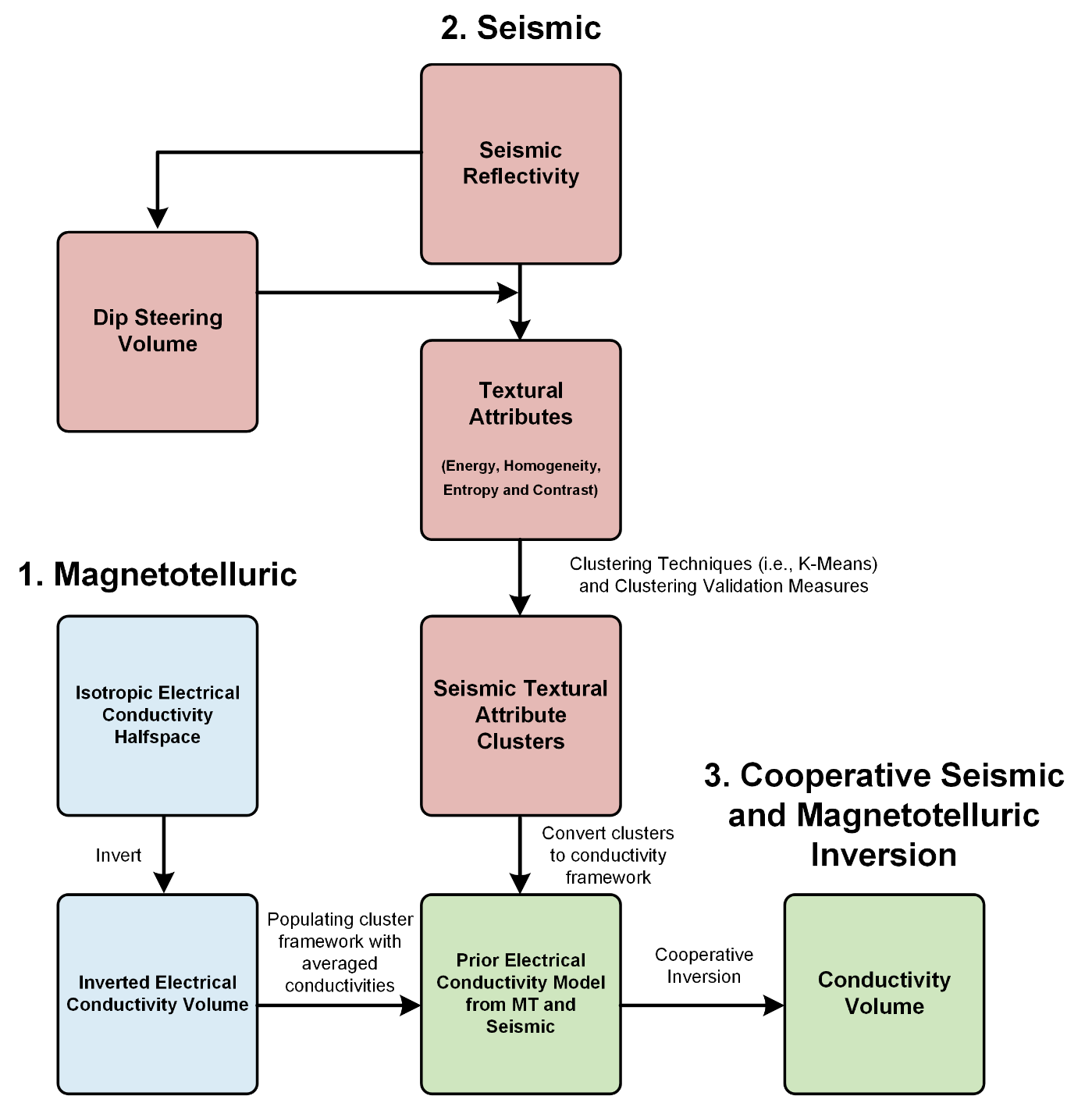


**Figure S2**. A schematic for seismic texture domaining combined with cooperative inversion of magnetotelluric and seismic data.

# References

1 Koivisto, E., Malehmir, A., Heikkinen, P., Heinonen, S. & Kukkonen, I. 2D reflection seismic investigations at the Kevitsa Ni-Cu-PGE deposit, northern Finland. *Geophysics* **77** (2012).

2 Malehmir, A. *et al.* 3D reflection seismic imaging for open-pit mine planning and deep exploration in the Kevitsa Ni-Cu-PGE deposit, northern Finland. *Geophysics* **77** (2012).

3 Malehmir, A. *et al.* A review of reflection seismic investigations in three major metallogenic regions: the Kevitsa Ni–Cu–PGE district (Finland), Witwatersrand goldfields (South Africa), and the Bathurst Mining Camp (Canada). *Ore Geology Reviews* **56**, 423-441 (2014).

4 Takam Takougang, E., Harris, B., Kepic, A. & Le, C. V. A. Cooperative joint inversion of 3D seismic and magnetotelluric data: With application in a mineral province. *Geophysics* **80**, 1-13, doi:10.1190/GEO2014-0252.1 (2015).

5 Le, C. V. A., Harris, B. D., Pethick, A. M., Takam Takougang, E. M. & Howe, B. Semiautomatic and Automatic Cooperative Inversion of Seismic and Magnetotelluric Data. *Surveys in Geophysics* **37**, 845-896, doi:10.1007/s10712-016-9377-z (2016).

6 Hillis, R. Coiled tubing drilling and real-time sensing-Enabling ‘prospecting drilling’in the 21st Century? Minerals keynote paper. *ASEG Extended Abstracts* **2015**, 1-1 (2015).

7 Hillis, R. *et al.* Coiled tube drilling and real-time sensing–enabling prospective drilling in the 21st century: Society of Economic Geologists Special Publication. (2014).

8 Le, C. V., Harris, B. D. & Pethick, A. M. Magnetotelluric inversion, carbonaceous phyllites and an ore zone: Kevitsa, Finland. *ASEG extended abstracts* **2016**, 1-5 (2016).

9 deGroot‐Hedlin, C. & Constable, S. Occam’s inversion to generate smooth, two‐dimensional models from magnetotelluric data. *Geophysics* **55**, 1613-1624, doi:10.1190/1.1442813 (1990).

10 Egbert, G. D. & Kelbert, A. Computational recipes for electromagnetic inverse problems. *Geophysical Journal International* **189**, 251-267, doi:10.1111/j.1365-246X.2011.05347.x (2012).

11 Kelbert, A., Meqbel, N., Egbert, G. D. & Tandon, K. ModEM: A modular system for inversion of electromagnetic geophysical data. *Computers & Geosciences* **66**, 40-53 (2014).

12 Moorkamp, M. Integrating Electromagnetic Data with Other Geophysical Observations for Enhanced Imaging of the Earth: A Tutorial and Review. *Surveys in Geophysics*, 1-28 (2017).
